# Agency effects on the binding of event elements in episodic memory

**DOI:** 10.1177/17470218231203951

**Published:** 2023-10-31

**Authors:** Marcel R Schreiner, Arndt Bröder, Thorsten Meiser

**Affiliations:** 1 Department of Psychology, School of Social Sciences, University of Mannheim, Mannheim, Germany; 2 Institute of Psychology, University of Würzburg, Würzburg, Germany

**Keywords:** Episodic memory, binding, memory integration, agency, statistical modelling

## Abstract

Representing events in episodic memory in a coherent manner requires that their constituent elements are bound together. So far, only few moderators of these binding processes have been identified. Here we investigate whether the presence of an agentic element in an event facilitates binding. The results from six experiments provided no evidence for a facilitating effect of agency on the binding of event elements. In addition, binding effects were only found when event elements were presented simultaneously, but not when they were presented sequentially pairwise, contrary to previous findings. The results suggest that the presence of an agentic element in an event does not, or only to a very limited extent, contribute to the formation of coherent memory representations and that additional processes may be required when binding event elements across temporarily divided encoding episodes. These findings add to a growing body of research regarding moderators and processes relevant for the binding of event elements in episodic memory. Explanations of these findings and directions for future research are discussed.

Experienced events stored in episodic memory encompass multiple elements, such as persons, objects, locations, actions, and sensations (Tulving, 1972, 1983). To allow for coherent event representations, these elements need to be bound together in memory. Such binding processes are associated with a stochastic dependency of the retrieval of event elements, such that the successful retrieval of an event element is associated with an increased likelihood of successful retrieval of subsequent event elements (Arnold et al., 2019; Boywitt & Meiser, 2012a, 2012b; Bröder, 2009; Horner & Burgess, 2013, 2014; Horner et al., 2015; Joensen et al., 2020; Meiser & Bröder, 2002; Schreiner et al., 2023; Starns & Hicks, 2005, 2008). For example, when having experienced an event of a dog biting a cat and a sparrow, being able to successfully retrieve the dog or the association dog–cat should increase the chance of also successfully retrieving the other event elements (the cat and the sparrow) or the other possible associations (dog–sparrow and sparrow–cat). These stochastic dependencies were used as indicators of binding effects. We use the term binding effect when referring to the conceptual level and the term stochastic dependency when referring to the observational level. Although the structure in which different event elements are bound together may vary (Schreiner et al., 2023; see also Eichenbaum, 1999), such a stochastic dependency should be observed as long as higher-level memory representations are formed. For example, in the study by Schreiner et al. (2023), we found evidence for both integrated representations, in which event elements are bound into a unitary representation (cf. Damasio, 1989; Marr, 1971; Moll & Miikkulainen, 1997; Shohamy & Wagner, 2008; Tulving, 1983), and hierarchical representations, in which event elements are bound in a system of pairwise bindings, with elements being preferentially bound to specific types of elements (cf. Cai et al., 2016; Cohen & Eichenbaum, 1993; Eichenbaum, 1999; Healy & Caudell, 2019; Hommel et al., 2001; Moeller et al., 2019). Although in an integrated representation, event elements can be readily retrieved jointly with all other elements, in a hierarchical structure, some elements may not be directly linked or linked less strongly than others. Yet, even if event elements are not directly linked, their association can be reconstructed given their indirect link via other elements and thus, the relationship between these elements also contributes to a stochastic dependency of the retrieval of event elements. Despite the importance of binding processes for the formation and retrieval of episodic memory representations, research on moderators influencing these binding processes has been scarce.

Only a small number of studies investigated moderators of the binding of event elements, encompassing aspects of stimulus presentation, event structure awareness, and animacy. James et al. (2020) identified the modality of stimulus presentation and its dimensionality as potential moderators influencing the binding of event elements, with written (rather than pictorial) stimuli and unidimensional (rather than multidimensional) stimulus presentation facilitating binding processes. There is also some evidence that awareness regarding the structure of an event (e.g., the number and types of elements that make up an event) is important for successful binding (Kumaran & Ludwig, 2013; Morton et al., 2020; Schreiner et al., 2023). In addition, animacy plays a major role in human memory (Nairne et al., 2013, 2017). According to the animacy effect, words representing animate entities are retrieved more likely than words representing inanimate entities (Li et al., 2016; Nairne et al., 2013; VanArsdall et al., 2015). Animate entities are living things that are capable of independent movement and can change direction without warning (Bonin et al., 2015). The animacy effect on memory has been found across a variety of test formats, including free recall (Bonin et al., 2015; Leding, 2019; Li et al., 2016; Madan, 2021; Nairne et al., 2013; Popp & Serra, 2016), recognition (Bonin et al., 2014; Leding, 2020; VanArsdall et al., 2013), and judgements of learning (DeYoung & Serra, 2021; Li et al., 2016). Results using cued recall tests have been mixed, with some studies finding the animacy effect (DeYoung & Serra, 2021; Laurino & Kaczer, 2019; VanArsdall et al., 2015) and others finding an opposite effect (Kazanas et al., 2020; Popp & Serra, 2016). Beyond enhancing memory performance, we previously found first evidence suggesting that animacy also facilitates the binding of event elements in episodic memory (Schreiner et al., 2023).

A potential explanation for the facilitating effect of animacy on the binding of event elements may be that the presence of an animate element provides a potential agent in an event. The concepts of animacy and agency are highly confounded, since animates are typically agentic. Thus, previously observed effects of animacy on binding (Schreiner et al., 2023) may actually be driven by agency. If this is the case, similar effects should be observed when event elements are equated regarding their animacy, but differ regarding their agency. Agency may be considered a property of animacy and may, in principle, also extend to inanimate elements (Johnson & Barrett, 2003; Lowder & Gordon, 2015). Animacy itself may thus be only one of several factors driving agency. Another factor may be the actual performance of an action. For example, an animal performing an action may be perceived as being more agentic than a passive animal or an animal that is the recipient of an action. Thus, agency may be a more proximate explanation for effects of animacy on binding. Agency can be defined as “acting or having the capacity to act autonomously in a given environment” (Suitner & Maass, 2016, p. 248; see also Hitlin & Elder, 2007) and is associated with concepts such as control over an action, dominance, competence, activity, and efficiency (Abele et al., 2008; Abele & Wojciszke, 2007; Bandura, 1989; Wojciszke et al., 2009). Agency plays an important role in status perception and stereotype formation (Carrier et al., 2014; Conway et al., 1996; Koch et al., 2016). Research on effects of agency in relation to memory has been scarcer. Most studies focused on agency on the participants’ side. For example, Woike et al. (1999) and Woike and Polo (2001) found the agency orientation of participants to affect the content and structure of recalled autobiographical memories. Agentic-motivated individuals reported memories that were more congruent with their motives and structured them using more differentiation. Jainta et al. (2022) found effects of agency during encoding on episodic memory. When encoding different episodes by imitating or merely observing videos showing short stories, participants exhibited stronger hippocampal responses to expectation violations when they were actors rather than observers in the episode. Self-performed episodes were also found to be remembered better than observed ones (Hornstein & Mulligan, 2001). In this study, participants heard action phrase, some of which they enacted themselves and some of which were enacted by the experimenter and merely observed by the participants. Hornstein and Mulligan (2001) found a consistent recall advantage for self-performed compared with observed actions (see also Engelkamp, 1986; Roberts et al., 2022 for further information on the enactment effect on memory). Huffman and Brockmole (2020) and Wen and Haggard (2018) found a bias in visual attention for objects that were under the participants’ control, thus invoking a sense of agency. In these studies, participants could control the movement of circles on a screen to varying degrees, whereas other circles moved randomly. Huffman and Brockmole (2020) and Wen and Haggard (2018) found reduced reaction times for targets under the participants’ control compared with targets not under their control or under their control to a lesser extent in a visual search task. Stimuli over which one feels a sense of agency are also remembered better than stimuli for which this is not the case (Hon & Yeo, 2021). In this study, participants were presented words to which participants reacted with self-initiated and -decided key presses. The words then moved in the direction of the key press (congruent trials) or the opposite direction (incongruent trials). In a later surprise recognition test, participants performed better for congruent compared with incongruent trials. Finally, Ruiz et al. (2023) found participants’ agency to facilitate associative memory and the binding of event elements into coherent memory representations. In their study, participants could either freely choose a door or were asked to choose a highlighted door for contestants in a game show to receive a prize. Ruiz et al. (2023) found better memory for contestants, as well as better memory for contestant–door and contestant–prize associations, when participants could freely choose the door. Their results also suggest that the different elements were stored in a more integrated manner if participants had agency over the trial. Regarding the agency of (external) stimuli, Walker and Keller (2019) found a processing advantage for faces with attractive, likable, and agentic traits. Specifically, participants exhibited faster self-recognition of their own faces, when their facial characteristics were altered to appear more attractive, likable, and agentic and also preferentially selected faces with these kinds of alterations as representing their own faces best. In addition, a major principle in the organisation of object vision is a graded distinction between animate and inanimate entities in the ventral temporal cortex (an animacy continuum, Connolly et al., 2012; Sha et al., 2015; Thorat et al., 2019), to which agency is an important contributor (Haxby et al., 2020; Thorat et al., 2019). In this research, we investigate whether agency as part of the stimulus facilitates the binding of event elements in episodic memory. Considering that animacy effects in memory are commonly explained by survival-relatedness, originating from selective pressure on our ancestors (e.g., animate entities are potential prey or opponents, Nairne et al., 2007, 2008, 2013), a similar reasoning may be applied to agency. For example, agentic entities may be particularly dangerous opponents.

We investigated whether the presence of an agentic element in an event facilitates the binding of event elements in six experiments. In Experiments 1–3, event elements were presented sequentially pairwise (cf. Horner & Burgess, 2014; Horner et al., 2015), whereas they were presented simultaneously (cf. Horner & Burgess, 2013) in Experiments 4–6. The sequential pairwise presentation provides a very strict test of binding, because coherent memory representations need to be formed across several temporarily divided encoding episodes. Thus, binding effects are indicative of a pure form of binding in memory, because they are less likely to occur due to covariations in perceptual variables. For example, temporal fluctuations in visual attention would co-occur with the presentation of all event elements given simultaneous presentation, which may increase the chance of later retrieving all or none of the event elements. Given sequential presentation, such fluctuations would not affect all event elements to the same extent, since their presentation is distributed across different encoding episodes. However, given the reduced temporal contiguity of encoding episodes compared with simultaneous presentation of event elements, this form of event presentation deviates from how events are naturally experienced. Binding effects given simultaneous presentation of event elements tend to be more robust (see James et al., 2020). Because we did not find stochastic dependencies in event element retrieval, which we used as indicators for binding effects, after sequential learning in Experiments 1–3, the following Experiments 4–6 employed a simultaneous presentation mode to test for effects of agency on binding. There we found significant dependencies, but the results provided no evidence for facilitating effects of agency on the binding of event elements.

## Experiments 1–3

In Experiments 1–3, we investigated whether the presence of an agentic element in an event facilitates binding, using the separated encoding paradigm (Horner & Burgess, 2014; Horner et al., 2015; see also Schreiner et al., 2023), in which each pairwise association in an event is presented separately during encoding. That is, for an event consisting of three elements (A, B, and C), the presentation of the event is distributed across three trials, in each of which two of the three elements are presented, therefore presenting the associations A–B, A–C, and B–C, while the presentation of trials is interleaved with trials referring to other events. However, we did not observe any binding effects in these experiments and thus, the experiments were not suitable for testing the moderating role of agency on binding processes (there was nothing to be moderated). We only document the rationale for Experiments 1–3 briefly in the main article and report on the details of the experiments in the Supplementary Appendix. In the main article, we focus on Experiments 4–6, because in these experiments we did observe binding effects and could thus investigate whether binding processes are facilitated by agency.

We used a linguistic agency manipulation in the experiments. In sentences containing interpersonal action verbs (e.g., *Paul hits Ted*.), the agent tends to be the grammatical subject, whereas the recipient of an action tends to be the grammatical object (Kasof & Lee, 1993). Consequently, grammatical subjects are perceived as being more agent-like than grammatical objects (Kako, 2006) and, for action verbs, greater causal weight is given to the agent than to the recipient (Brown & Fish, 1983; Kassin & Lowe, 1979). For example, animacy tends to be a strong predictor of subject assignment (Prentice, 1967) and animate referents are usually agentic. In addition, there is an influence of transitivity. Fausey and Boroditsky (2010) found linguistic framing to influence participants’ judgements of blame and financial liability. People who read transitive agentive frames (e.g., *Timberlake ripped the costume*.) allocated higher blame and financial liability than people who read intransitive non-agentive frames (e.g., The costume ripped.). A potential mechanism for these effects may be conceptual accessibility, which describes the ease of activation or retrieval of mental representations of a potential referent (Bock & Warren, 1985). Both animate and agentic referents are more conceptually accessible than inanimate or recipient referents (Gleitman et al., 2007; Prat-Sala & Branigan, 2000; Rissman et al., 2019). Another explanation may be that agents (and grammatical subjects) are more salient than recipients (and grammatical objects), particularly in third-person interpersonal action sentences (Kasof & Lee, 1993; Prat-Sala & Branigan, 2000).

We thus created agentic event elements by placing them as grammatical subjects in transitive active sentences (e.g., The dog grabs the eagle.), whereas the non-agentic elements were placed as the grammatical objects. If the sentence contained only non-agentic elements, we used passive sentences (e.g., The dog and the eagle are being grabbed.), in which the grammatical subject is not the agent of the event (Kako, 2006). Events consisted of three elements. Therefore, for example, the following sentences were presented for an event in the agency condition consisting of a dog, an eagle, and an ant: *The dog grabs the eagle*., *The dog grabs the ant*., and *The dog and the eagle are being grabbed*. In the non-agency condition, the same event would be presented as: *The dog and the eagle are being grabbed., The dog and the ant are being grabbed*., and *The eagle and the ant are being grabbed*. The use of active verb forms in the agency condition is an additional component that should increase perceived agency, whereas the use of passive verb forms should diminish it (see Henley et al., 1995; see also Halicki et al., 2021). We expected to find a stronger stochastic dependency of the retrieval of event elements for events with an agentic element (i.e., with a subject acting on another event element) than for events without an agentic element (Hypothesis 1). All experiments were preregistered (Experiments 1 and 2: https://osf.io/kts8p, Experiment 3: https://osf.io/vhmt4).

The absence of binding effects in Experiments 1–3 contrasts with previous findings of binding effects using the separated encoding paradigm (Bisby et al., 2018; Horner & Burgess, 2014; Horner et al., 2015; Joensen et al., 2020; Schreiner et al., 2023). However, these studies used individual words or images as stimuli, whereas stimuli were embedded in sentences in our experiments. Presenting individual stimuli gives participants the opportunity to freely associate them and may reduce the prevalence of nonsensical scenes, thus facilitating the formation of coherent memory representations. Therefore, it may be the case that the presentation of sentences (a more prestructured presentation format) in the separated encoding paradigm hinders the formation of coherent memory representations. Since our agency manipulation relies on this presentation format, the separated encoding paradigm may not be suitable to investigate effects of agency on the binding of event elements in this research. Experiment 2 was additionally designed to investigate the structure in which event elements are bound (cf. Horner & Burgess, 2014; Horner et al., 2015; Schreiner et al., 2023), but yielded uninformative results concerning this question. This was again due to the lack of observed binding effects. In Experiment 4, we switched to the simultaneous encoding paradigm (Horner & Burgess, 2013), in which all event elements are presented simultaneously. This paradigm tends to yield more robust binding effects (see James et al., 2020).

## Experiment 4

In Experiment 4 we investigated whether there is a stronger stochastic dependency of the retrieval of event elements for events with an agentic than for events without an agentic element (Hypothesis 1), but changed the experimental paradigm to the simultaneous encoding paradigm (Horner & Burgess, 2013). In the simultaneous encoding paradigm, all event elements are presented simultaneously in a single learning trial, instead of being presented sequentially pairwise across different learning trials, as is the case in the separated encoding paradigm (Horner et al., 2015; Horner & Burgess, 2014). Given that the presentation of event elements embedded in sentences describing scenes seemed to hinder the formation of coherent memory representations in the separated encoding paradigm, this problem may not occur in the simultaneous encoding paradigm, in which it is no longer necessary to build coherent memory representations across temporarily divided encoding episodes. The experiment’s design, hypothesis, and analysis plan were preregistered at https://osf.io/q5tme.

### Methods

#### Participants

Participants were recruited via Prolific (https://www.prolific.co/) and received a compensation of £2.50 (£7.50/hr). They were prescreened to be native German speakers, to not conduct the study on a smartphone, and to not have participated in Experiment 3. An a priori power analysis with simulated data for detecting a medium difference between conditions (difference in event-specific trait variances of 1 according to the statistical procedure [see below], cf. Glas et al., 2000; Wang et al., 2002 assumed baseline event-specific trait variance of 2)^1^ with 80% power using one-tailed testing yielded a desired sample size of 200 participants (100 participants per between-subjects condition). Due to the potential necessity of some data exclusion, we increased the desired sample size by 20% and collected data from 241 participants.^2^ All participants provided online informed consent for their participation and publication of their data. Two participants were excluded because they suggested their data should not be used for the study (e.g., due to tiredness).^3^ Thus, the final sample consisted of 122 participants in the agency condition (59 [48%] female, 1 [1%] non-binary, 43 [35%] students), with an average age of 31.3 years (*SD* = 10.7, range = 18–62), and 117 participants in the non-agency condition (53 [45%] female, 43 [37%] students), with an average age of 32.0 years (*SD* = 11.3, range = 18–72).^4^

#### Design

The experiment employed a one-factorial (agency condition: agency vs non-agency) between-subjects design. In the agency condition, one event element served as the agent and was placed as the grammatical subject in active sentences (e.g., The dog grabs the eagle and the ant.). In the non-agency condition there was no agent and only passive sentences were used (e.g., The dog, the eagle, and the ant are being grabbed.). Participants were randomly assigned to the experimental conditions.

#### Material

Stimuli consisted of 72 German nouns representing three different animal types: 24 mammals (e.g., dog, partly taken from the study by Schreiner et al., 2023), 24 birds (e.g., eagle), and 24 insects^5^ (e.g., ant). In addition, 24 verbs (a subset of the ones of Experiment 1) were used. An additional nine nouns (three of each type) and three verbs were used as primacy buffers for preventing primacy effects. Using three types of animals avoids confounding with animacy (cf. Schreiner et al., 2023), assuming that animacy is constant across the different animal types. The same set of stimuli was used for the agency and non-agency condition. Using the stimuli, we randomly created 24 events and 3 primacy buffer events for each participant, each consisting of a mammal, a bird, an insect, and a verb. Events were randomly assigned to the two experimental conditions, resulting in 12 events per condition and 3 primacy buffer events per condition, which were presented first.

#### Procedure

**Figure 1.**
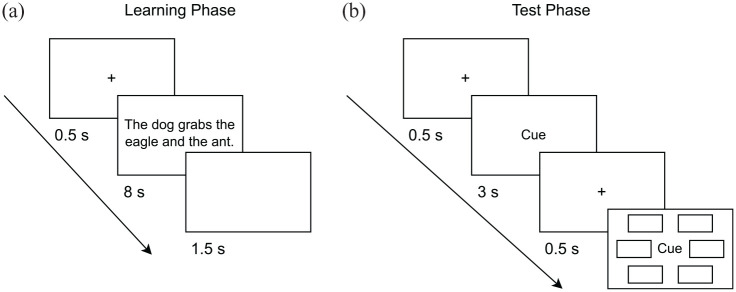
Experimental procedure of Experiment 4. (a) Exemplary learning trial in the agency condition and (b) schematic depiction of a test trial.

The experiment was conducted online and implemented using lab.js (Henninger et al., 2022). Data collection was managed by JATOS (Lange et al., 2015). The procedure was based on the simultaneous encoding paradigm (Horner & Burgess, 2013). In the learning phase of the experiment, participants were presented a sentence containing all three event elements^6^ and the verb associated with the event in each trial. Participants were instructed to imagine the scenes described by the sentences and make them seem as vivid as possible. There was one learning trial (i.e., sentence) for each event. In the agency condition there was one agent (grammatical subject) and two recipients (grammatical objects) in an active sentence (e.g., *The dog grabs the eagle and the ant*.). Each stimulus type (i.e., mammal, bird, or insect) served as the agent equally often across events. Thus, whether the agent was a mammal, a bird, or an insect was counterbalanced across events. In the non-agency condition there were three non-agentic elements in a passive sentence (e.g., *The dog, the eagle, and the ant are being grabbed*.). The sentence positions of the non-agentic elements were randomised (this concerns the two elements serving as grammatical objects in the agency condition and all three elements in the non-agency condition). Event elements in the agency condition were defined in terms of whether they were the agent or one of the non-agents in an event, yielding the associations *agent–non-agent_1_, agent–non-agent_2_, and non-agent_1_–non-agent_2_*. Event elements in the non-agency condition were defined in terms of their stimulus type, yielding the associations mammal–bird, mammal–insect, and bird–insect. Each trial consisted of a 0.5-s fixation cross, an 8-s sentence presentation, and a 1.5-s blank screen (see Figure 1a). Primacy buffers were presented at the beginning of the learning phase to prevent primacy effects and were not used for the later test phase. In addition, participants were asked to click on a continue button after the primacy buffer trials and after 50% of learning trials to keep them engaged during the learning phase.^7^ After the learning phase, participants conducted a filler task in which they had to solve randomly generated math problems for 3 min to avoid recency effects.

In the subsequent test phase, participants performed an incidental cued recognition forced-choice task. In each test trial, participants were first presented a 0.5-s fixation cross, followed by a 3-s presentation of the cue word (one of the event elements shown in the learning phase),^8^ displayed in the screen centre. After another 0.5-s fixation-cross, the cue word was again displayed in the screen centre and six response alternatives were displayed in a hexagonal array around it (see Figure 1b). Participants had to select the target response alternative that belonged to the same event as the cue word. All response alternatives were of the same stimulus type (e.g., all mammals) and distractors were randomly drawn from other events.^9^ Given three associations per event, a total of six tests are possible, considering that each element in an association could serve as the cue in one test of the association and as the target in another test. To avoid testing effects, we tested each association in only one direction (i.e., with an element serving as either cue or target). We also imposed the constraint that each element type serves as cue and target equally often across events. This was accomplished by forming two sets of possible cue–target pairs for testing associations. For example, in the non-agency conditions, these were: (1) mammal–bird, bird–insect, and insect–mammal and (2) mammal–insect, insect–bird, and bird–mammal. The selected set of cue–target pairs was counterbalanced across events. This resulted in three test trials per event, with each association of an event being tested. The test phase consisted of three blocks. For each participant and event, the three tested associations were randomly assigned to the three blocks, such that one association per event was tested in each block. Within each block, the order of test trials was randomised for each participant.

#### Data analysis

All analyses were conducted in the R Programming Environment (R Core Team, 2021) and we used the R packages *papaja* (version 0.1.1, Aust & Barth, 2022) and *tinylabels* (version 0.2.3, Barth, 2022) for reporting. We used the conventional significance level of α=0.05 for the analyses. For the exploratory analysis of memory performance we computed Bayes factors in favour of an effect. Thus, a Bayes factor > 1 is in favour of an effect, whereas a Bayes factor < 1 is in favour of the absence of an effect (see Jeffreys, 1961).

##### Exploratory analysis of memory performance

For an exploratory analysis of memory performance, we used Bayesian generalised linear mixed models with a logit link function (Goldstein, 2011; Rouder & Lu, 2005). Test trial outcomes (i.e., whether a correct response was given by selecting the target or an incorrect response was given by selecting a distractor in the cued recognition test) served as a binary dependent variable. Thus, individual trial information, rather than aggregate information, was entered into the model (see Hoffman & Rovine, 2007). We investigated effects of agency condition, association^10^, and the interaction. We also included random person intercepts to account for repeated measurement and random item intercepts.^11^ Because, in the agency-condition, association refers to the agent or non-agent status of the cue and the target, whereas in the non-agency condition it refers to stimulus type (i.e., the factor association has different levels in the agency and non-agency condition), we equated associations across agency conditions to jointly include them in the models. We equated corresponding factor levels, thus coercing the associations *agent–non-agent_1_ and mammal–bird, agent–non-agent_2_ and mammal–insect, and non-agent_1_–non-agent_2_* and bird–insect into a common factor level, respectively. For the main effects, this assumes that the respective coerced associations are equivalent, which may not necessarily be the case, although the way associations were equated may account to some extent for possible differences in perceived animacy or agency of different animal categories (see Connolly et al., 2012; Sha et al., 2015; Thorat et al., 2019). However, differential effects of associations within agency conditions would result in an interaction of agency condition and association and can therefore be investigated. To assess the influence of each factor, we fit several models with different predictors and compared them with a baseline model. To investigate the main effects, we compared a model including the respective predictor (condition or association) with a null model including only fixed and random person intercepts and random item intercepts. To investigate the interaction, we compared the full model containing both main effects and the interaction with a model including both main effects but no interaction. We then computed Bayes factors in favour of an effect $\left({\mathrm{BF}}_{10}\right)$ for each predictor.

Models were fit and Bayes factors were computed using the R package *brms* (version 2.19.0, Bürkner, 2017, 2018) using a standard normal prior for fixed effects and a half Student’s-*t* prior with three degrees of freedom (the default) for random effects. As a robustness check, we also fit the models with less informative normal priors (*SD* = 4) and more informative normal priors (*SD* = 0.25) for fixed effects and report the Bayes factors computed on the basis of these models in brackets behind the Bayes factors computed on the basis of models with standard normal priors for fixed effects. Models were fit with 4 Markov chains and 30,000 iterations per chain, the first 15,000 of which were used as burnin iterations. In addition, we report the model estimates and 95% credible intervals based on the full model with standard normal priors for fixed effects.

To test whether memory performance in the agency and non-agency condition differed from chance (i.e., 1/6), we conducted one-sample Wilcoxon signed-rank tests (one-tailed testing). We used a nonparametric test because Shapiro–Wilk tests and visual inspection of quantile–quantile plots indicated significant deviations from normality for the distributions.

##### Dependency analysis

**Figure 2.**
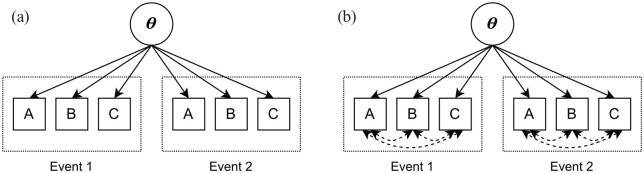
A latent variable model with a latent trait θ and six items that belong to two different events. (a) Depicts the case where binding effects are absent and all item residual correlations are zero and (b) depicts the case where binding effects are present and item residual correlations within an event deviate from zero.

For modelling the stochastic dependency of the retrieval of event elements as an indicator of binding effects, we used the approach by Schreiner et al. (2023) and Schreiner and Meiser (2023), which is based on item response theory (IRT, Lord, 1980; Lord & Novick, 1968). Assume a latent variable model like the one depicted in Figure 2. The depicted model contains one latent trait θ , which reflects memory performance, and six manifest item responses (A, B, and C), which reflect test trials probing specific event elements. In addition, items refer to two different events and can be clustered accordingly. The IRT model we used as the foundation for the dependency analysis assumes local independence, meaning that the latent person trait accounts for all inter-item relationships (de Ayala, 2009; Lazarsfeld & Henry, 1968). Therefore, item responses are independent when accounting for the effect of the latent trait. This case is depicted in Figure 2a, where there are no connections between items beyond their indirect connection via the latent trait. In other words, all item residual correlations are zero in this case. Given the presence of binding effects, however, these would induce additional event-specific effects. These event-specific effects would induce relations between items beyond the latent trait. This case is depicted in Figure 2b, where there are now additional connections between items. Note that these connections are clustered within an event, because we typically assume no binding across different events. In other words, given binding effects, item residual correlations between item pairs referring to the same event deviate from zero. These item residual correlations reflect the stochastic dependency of the retrieval of event elements. Therefore, the modelling approach essentially quantifies violations of the model assumption of local independence, while considering the systematic nature in which these violations should occur, given binding effects.

We fit a simplified three-parameter logistic IRT model (A. Birnbaum, 1968) to the data of each agency condition (because agency was manipulated between-subjects), with discrimination parameters fixed to 1, since events were randomly generated, and guessing parameters fixed to the stochastic guessing probability of 1/6 given six response options in the cued recognition test:

$$P\left(u_{\mathrm{ij}}=1\right)=\frac{1}{6}+\frac{5}{6}\frac{e^{\theta_{i}-\beta_{j}}}{1+e^{\theta_{i}-\beta_{j}}}$$

It models the probability of person *i* to give a correct response to item *j* given a latent person trait θ , representing memory performance in the current application, and item difficulty β .^12^ Note that the model equation is a logistic function, therefore, accounting for the binary nature of the items (i.e., test trial outcomes in the cued recognition test). Based on this model, we computed item residual correlations using the $Q_{3}$ statistic (Yen, 1984) with a bias correction (Yen, 1993) applied. The dependency measure *D* is then computed by contrasting the mean residual correlation between item pairs referring to the same event (*kk*′) with the mean residual correlation between item pairs referring to different events (*ll*′):

$$D=\frac{1}{K}\sum_{k>k^{\prime }}Q_{3}^{kk\prime }-\frac{1}{L}\sum_{l>l^{\prime }}Q_{3}^{ll\prime }$$

with *K* being the total number of item pairs referring to the same events and *L* being the total number of item pairs referring to different events. As described above, given binding effects, item residual correlation should deviate from zero for item pairs referring to the same event (the first part of the equation), but should be close to zero for item pairs referring to different events (the second part of the equation), as we would not expect binding effects between different events. We nevertheless contrast the mean residual correlations between item pairs referring to the same event with the mean residual correlations between item pair referring to different events to control for baseline dependencies in empirical data and isolate the dependency that is specifically due to items being associated with a common event. This also makes *D* robust against model misspecification, since this would affect both the item residual correlations referring to the same event and item residual correlations referring to different events.

Given our reliance on the $Q_{3}$ statistic (Yen, 1984) for estimating item residual correlations reflecting the stochastic dependency of the retrieval of event elements, for determining whether dependencies or differences in dependency are significant, we need to consider the sampling distribution of the $Q_{3}$ statistic. However, the sampling distribution of $Q_{3}$ , and thus also the one of *D*, is unknown (Chen & Thissen, 1997). Therefore, the approach uses parametric bootstrapping for obtaining *p* values (see Schreiner et al., 2023; Schreiner & Meiser, 2023). In parametric bootstrapping, a simulated distribution of a statistic is generated by repeatedly generating data from estimated parameters under the assumption that the data-generating model is true. To test whether dependency estimates differed from zero, we repeatedly sampled from the model in equation (1), which assumes no dependency (i.e., assumes that the local independence assumption holds), using the empirically estimated item parameters. Person parameters were drawn from a normal distribution with a mean of zero and the empirically estimated latent trait variance. We then computed *D* for each sample and condition and used the resulting distributions for computing two-tailed *p*-values and standard errors. To test whether dependency estimates differed between experimental conditions, we repeatedly sampled from a bifactor IRT model (see Gibbons & Hedeker, 1992; Wainer & Wang, 2000), which extends the model in equation (1) by additional event-specific latent traits that exert their influence via their variance, thus inducing stochastic dependencies between items of the same event (for model equations, see Schreiner & Meiser, 2023). Item parameters were empirically estimated by fitting a bifactor IRT model to the data. Person parameters were drawn from a multivariate normal distribution with means and covariances of zero and empirically estimated variances. Because events were randomly generated, we set equality constraints on the event-specific trait variances within conditions when fitting the bifactor model. When sampling from the model, we set the event-specific trait variances of both conditions equal to the one of the condition with the smaller event-specific trait variance so the model assumes no difference in dependency between conditions. We then computed the difference of *D* between conditions for each sample and used the resulting distribution for computing a one-tailed *p*-value and the standard error.

The R package *mirt* (version 1.35.1, Chalmers, 2012) and adapted functions from the package *sirt* (version 3.9-4, Robitzsch, 2020) were used for the dependency analysis. The package *SimDesign* (version 2.8, Chalmers & Adkins, 2020) was used for conducting the parametric bootstraps. We used 1,000 bootstrap samples for each bootstrap (cf. Davison & Hinkley, 1997).

Fitting the bifactor IRT models yielded some extreme estimates for item parameters that, when being used as input for the parametric bootstrap for testing differences between the experimental conditions, caused item responses in the simulated data to have no variance. This prevented the estimation of item parameters for these items (cf. Snodgrass & Corwin, 1988) and consequently the computation of the dependency measure in the bootstrap. Instead of adjusting these extreme item parameters (four parameters [6%] in the model for the agency and non-agency condition, respectively) by a fixed constant (cf. Snodgrass & Corwin, 1988), we used a model-based approach to substitute them with random values drawn from the empirical distribution of the remaining parameters, using the *remp* function from the package *fishmethods* (version 1.11-3, Nelson, 2022).

### Results

#### Memory performance

**Figure 3.**
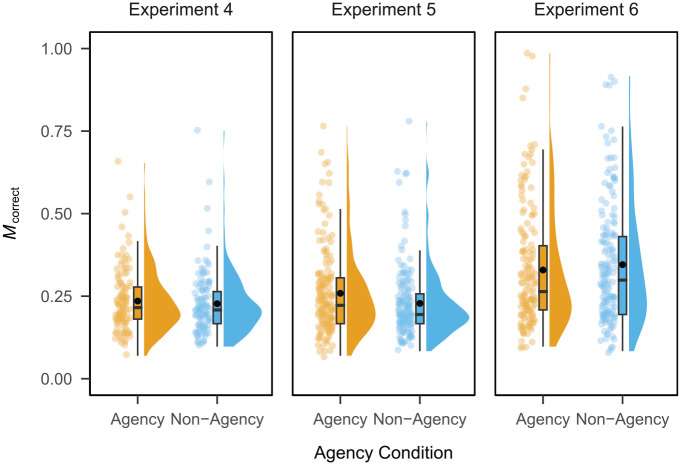
Raincloud plot depicting the proportion of correct responses per participant by agency condition in Experiments 4, 5, and 6. Black dots depict the mean across participants.

On average, the proportion of correct responses was *M* = 0.24 (*SD* = 0.42) in the agency condition and *M* = 0.23 (*SD* = 0.42) in the non-agency condition. Performance was significantly above chance in both the agency ( V=6,070.50 , p<.001 , *r* = .67) and non-agency ( V=5,380.00 , p<.001 , *r* = .64) condition. Figure 3 shows a raincloud plot (Allen et al., 2021) of the proportion of correct responses per participant. There was evidence against a main effect of condition ( ${\mathrm{BF}}_{10}=0.08$ [0.02, 0.32], β=−0.06 , *SE* = 0.09, 95% confidence interval [CI] = [–0.23, 0.11]), evidence against to no evidence for a main effect of association (depending on the choice of prior, ${\mathrm{BF}}_{10}=0.15$ [0.010, 2.21], agent–non-*agent_2_*/mammal–insect: β=0.03 , *SE* = 0.06, 95% CI = [–0.09, 0.16], *non-agent_1_–non–agent_2_*/bird–insect: β=−0.08 , *SE* = 0.06, 95% CI = [–0.20, 0.05]), and evidence against an interaction of condition and association ( ${\mathrm{BF}}_{10}=0.009$ [< 0.001, 0.12], non-agency × agent–non-*agent_2_*/mammal–insect: β=0.05 , *SE* = 0.09, 95% CI = [–0.13, 0.23], non-agency × *non-agent_1_–non-agent_2_*/bird–insect: β=0.00 , *SE* = 0.09, 95% CI = [–0.18, 0.18]).

#### Dependency

**Figure 4.**
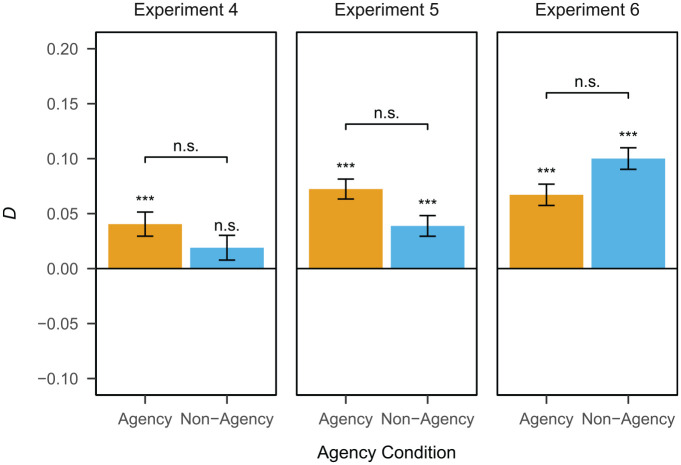
Dependency of the retrieval of event elements by agency condition in Experiments 4, 5, and 6. ***p < .001. n.s = non-significant. Error bars represent ± *SE*.

The dependency of the retrieval of event elements is shown in Figure 4. There was a significant positive dependency in the agency condition (*D* = 0.04, *SE* = 0.01, *p* < .001). The dependency in the non-agency condition was non-significant (*D* = 0.02, *SE* = 0.01, *p* = .12). Testing for a difference in dependency between conditions, the dependency in the agency condition was not significantly larger than the one in the non-agency condition ( $D_{\mathrm{diff}}=0.02$ , *SE* = 0.03, *p* = .22).

### Discussion

In Experiment 4, we tested Hypothesis 1, which states that there is a stronger stochastic dependency of the retrieval of event elements for events with an agentic element than for events without an agentic element. Contrary to Experiments 1–3, all event elements were presented simultaneously. The pattern of results, with a significant positive dependency in the agency condition and no significant dependency in the non-agency condition, is in favour of the hypothesis and suggests that the presence of an agentic element in an event facilitates the binding of event elements, leading to more coherent memory representations. However, the difference in dependency between conditions did not reach significance. Thus, Hypothesis 1 was only partially supported. It may be the case that the difference in dependency between conditions was smaller than anticipated and thus the study did not have enough power for reliably detecting the difference. In Experiment 5, we aimed to replicate the pattern of results and to detect also smaller differences between conditions by increasing the sample size.

## Experiment 5

Experiment 5 was a replication of Experiment 4 and we thus again investigated whether there is a stronger stochastic dependency of the retrieval of event elements for events with an agentic than for events without an agentic element (Hypothesis 1). In Experiment 5, we aimed at a higher power for detecting smaller differences between the experimental conditions. We also slightly increased the duration of the learning trials to improve memory performance. The experiment’s design, hypothesis, and analysis plan were preregistered at https://osf.io/g59uh.

### Methods

#### Participants

Participants were recruited via Prolific (https://www.prolific.co/) and received a compensation of £2.63 (£7.51/hr). They were prescreened to be native German speakers, to not conduct the study on a smartphone, and to not have participated in Experiments 3 and 4. An a priori power analysis (the same as in Experiment 4) for detecting a small to medium difference between conditions (difference in event-specific trait variances of 0.75 according to the statistical procedure, cf. Glas et al., 2000; Wang et al., 2002 assumed baseline event-specific trait variance of 2) with 80% power using one-tailed testing yielded a desired sample size of 300 participants (150 participants per between-subjects condition). Due to the potential necessity of some data exclusion, we increased the desired sample size by 20% and collected data from 360 participants. All participants provided online informed consent for their participation and publication of their data. The data of one participant were not transmitted due to a technical error. One participant was excluded because they processed less than five math problems during the filler task. Another six participants were excluded because they suggested their data should not be used for the study (e.g., due to not properly understanding the instructions). An additional participant was excluded because their data suggested that they interrupted the study for a long duration of about 9 min during the learning phase. Thus, the final sample consisted of 180 participants in the agency condition (98 [54%] female, 2 [1%] non-binary, 82 [46%] students), with an average age of 29.7 years (*SD* = 10.2, range = 18–70), and 171 participants in the non-agency condition (80 [47%] female, 3 [2%] non-binary, 75 [44%] students), with an average age of 31.2 years (*SD* = 10.3, range = 18–69).

#### Design, material, procedure, and data analysis

The experimental design, the stimuli, and the data analysis were identical to the ones of Experiment 4. Participants were randomly assigned to the experimental conditions. The experimental procedure was also identical to the one of Experiment 4 except that we increased the presentation duration of the sentences to 10 s and the duration of the blank screen to 2 s. Thus, each learning trial consisted of a 0.5-s fixation cross, a 10-s sentence presentation, and a 2-s blank screen.

### Results

#### Memory performance

On average, the proportion of correct responses was *M* = 0.26 (*SD* = 0.44) in the agency condition and *M* = 0.23 (*SD* = 0.42) in the non-agency condition. Performance was significantly above chance in both the agency ( V=12,913.00 , p<.001 , *r* = .68) and non-agency ( V=10,193.00 , p<.001 , *r* = .60) condition. The proportion of correct responses per participant is shown in Figure 3. There was, depending on the choice of prior, weak evidence against to weak evidence for a main effect of condition ( ${\mathrm{BF}}_{10}=\;1.17$ [0.30, 3.68]), but the 95% credible interval did not include zero, suggesting that memory performance was lower in the non-agency condition than in the agency condition ( β=−0.18 , *SE* = 0.08, 95% CI = [–0.34, –0.02]). There was, depending on the choice of the prior, evidence against to no evidence for a main effect of association ( ${\mathrm{BF}}_{10}=\;0.09$ [0.005, 1.35], *agent–non-agent_2_*/mammal–insect: β=0.05 , *SE* = 0.05, 95% CI = [–0.05, 0.15], *non-agent_1_–non–agent_2_*/bird–insect: β=−0.12 , *SE* = 0.05, 95% CI = [–0.22, –0.02]) and evidence against an interaction of condition and association ( ${\mathrm{BF}}_{10}=0.03$ [0.002, 0.31], non-agency × *agent–non-agent_2_*/mammal–insect: β=−0.03 , *SE* = 0.08, 95% CI = [–0.18, 0.11], non-agency × *non-agent_1_–non-agent_2_*/bird–insect: β=0.10 , *SE* = 0.08, 95% CI = [–0.05, 0.25]).

#### Dependency

The dependency of the retrieval of event elements is shown in Figure 4. There was a significant positive dependency in both the agency condition (*D* = 0.07, *SE* = 0.01, *p* < .001) and the non-agency condition (*D* = 0.04, *SE* = 0.01, *p* < .001). Testing for a difference in dependency between conditions, the dependency in the agency condition was not significantly larger than the one in the non-agency condition ( $D_{\mathrm{diff}}=0.03$ , *SE* = 0.02, *p* = .15).

### Discussion

In Experiment 5, we largely replicated the findings of Experiment 4, except that the dependency in the non-agency condition reached significance in Experiment 5. This may be due to increased power given the larger sample size. Dependencies in both conditions were also descriptively larger than in Experiment 4, which may be due to the increased learning trial duration, which may have given participants more time to form bindings during encoding. Although the difference in dependency between the agency and non-agency condition was descriptively larger than in Experiment 4, it did not reach significance, despite having a larger power for also detecting smaller effects in Experiment 5. Thus, Hypothesis 1 was not supported. However, descriptively, the results pointed in the expected direction, as was the case in Experiment 4. It may be the case that the relatively poor memory performance in the experiment conceals differences in dependency between the conditions (cf. Schreiner & Meiser, 2023). In Experiment 6, we made a number of changes to the experimental procedure to increase memory performance and account for possible confounds that may have been present in previous experiments.

## Experiment 6

In Experiment 6, we once again investigated whether there is a stronger stochastic dependency of the retrieval of event elements for events with an agentic than for events without an agentic element (Hypothesis 1). One goal of the experiment was to increase memory performance compared with the previous experiments. To this end, we presented each event (i.e., each sentence) twice during the learning phase and made the memory test intentional.^13^ Another goal was to account for a possible confound in previous experiments: In the agency condition of Experiments 4 and 5, sentences were always active, whereas sentences in the non-agency condition were always passive. To rule out possible confounding effects in Experiment 6, we adapted the agency manipulation. Although the procedure in the agency condition remained the same (i.e., we used transitive active sentences in which the agent performs an action described by a dynamic verb on the non-agentic elements, e.g., The dog grabs the eagle and the ant.), in the non-agency condition, we used intransitive active sentences in which the action of the “agent” was described by a stative verb next to the non-agentic elements (e.g., The dog stands next to the eagle and the ant.). Thus, active sentence structures were used in both the agency and non-agency condition and the agency manipulation relied on the transitivity of the sentence and the activity level of the verbs used (cf. Fausey & Boroditsky, 2010; Halicki et al., 2021; Henley et al., 1995; Lowder & Gordon, 2015). In addition, we included two attention checks to ensure good data quality. The experiment’s design, hypothesis, and analysis plan were preregistered at https://osf.io/c84mt.

### Methods

#### Participants

Participants were recruited via Prolific (https://www.prolific.co/) and received a compensation of £3.90 (£9.00/hr). They were prescreened to be native German speakers, to not conduct the study on a smartphone, and to not have participated in Experiments 2–4. As in Experiment 5, we collected data from 360 participants. All participants provided online informed consent for their participation and publication of their data. The data of three participants were not transmitted due to a technical error. We excluded 28 participants from the analyses because they did not pass both attention checks. Another participant was excluded because they processed less than five math problems during the filler task. Another three participants were excluded because they suggested their data should not be used for the study (e.g., due to misunderstanding instructions). Thus, the final sample consisted of 161 participants in the agency condition (84 [52%] females, 67 [42%] students), with an average age of 29.9 years (*SD* = 9.3, range = 18–67), and 164 participants in the non-agency condition (78 [48%] females, 1 [1%] non-binary, 78 [48%] students), with an average age of 28.9 years (*SD* = 9.1, range = 19–67).

#### Design

The design was identical to the one of Experiments 4 and 5, except that there was now also an “agent” in the non-agency condition, which was placed as the grammatical subject in intransitive active sentences. Participants were randomly assigned to the experimental conditions.

#### Material

Stimuli were the same as in Experiments 4 and 5, except that there were two different sets of verbs used in the agency and non-agency condition. The verbs used in the agency condition were dynamic and the same as the ones used in previous experiments.^14^ The ones used in the non-agency condition were a new set of stative verbs. In a pilot study (*N* = 20), we asked participants to rate each verb on the dimensions agency, activity, and emotionality using a visual analogue scale ranging from 0 (*very unagentic/very inactive/very negative*) to 100 (*very agentic/very active/very positive*).^15^ An analysis using mixed linear models (fit using the packages *lme4*, version 1.1-27.1, Bates et al., 2015; and *lmerTest*, version 3.1-3, Kuznetsova et al., 2017) revealed that the verbs used in the non-agency condition were rated as less agentic, $M_{\mathrm{agency}}=58.94$ , $M_{\mathrm{non-agency}}=39.81$ , *b* = –19.13, *SE* = 1.60, *t*(1,059.00) = –11.99, *p* < .001, and less active, $M_{\mathrm{agency}}=68.13$ , $M_{\mathrm{non-agency}}=\;31.48$ , *b* = –36.65, *SE* = 1.47, *t*(1,059.00) = –24.99, *p* < .001, but were not rated differently in emotionality, $M_{\mathrm{agency}}=17.47$ , $M_{\mathrm{non-agency}}=17.12$ , *b* = –0.35, *SE* = 0.93, *t*(1,059.00) = –0.38, *p* = .71, than the verbs used in the agency condition.^16^

#### Procedure and data analysis

The procedure was identical to the one of Experiment 5 with the following exceptions: Participants were informed at the beginning of the learning phase that their memory regarding the scenes described by the presented sentences will later be tested, thus making the memory test intentional. In the learning phase, each event was presented twice (except for primacy buffer events). Thus, after all events were presented, they were again presented in randomised order. In the non-agency condition, intransitive active sentences were used in which the “agent” was described to perform an action described by a stative verb next to the non-agentic elements (e.g., *The dog stands next to the eagle and the ant*.). The procedure in the agency condition remained the same and thus, transitive active sentences were used in which the agent performed an action described by a dynamic verb on the non-agentic elements (e.g., *The dog grabs the eagle and the ant*.). The “agent” served as the grammatical subject in both the agency and non-agency condition and it was counterbalanced across events whether the “agent” was a mammal, a bird, or an insect in both conditions. In addition, the experiment included two attention checks (reinstating those used in Experiments 1 and 2). After 50% of learning trials (not counting primacy buffers), participants were asked to click on a continue button within 10 s and after 50% of test trials they were asked to select the top left response option. Data analysis was identical to the one of Experiment 5, except that there were only two types of associations (agent–non-agent and non-agent–non-agent) in both experimental conditions. Post-hoc pairwise comparisons were conducted using the package *emmeans* (version 1.8.6, Lenth, 2022). We considered a difference to be substantial if the 95% credible interval (highest posterior density interval) does not include zero.

### Results

#### Memory performance

On average, the proportion of correct responses was *M* = 0.33 (*SD* = 0.47) in the agency condition and *M* = 0.35 (*SD* = 0.48) in the non-agency condition. Performance was significantly above chance in both the agency ( V=11,578.00 , p<.001 , *r* = .80) and non-agency ( V=12,345.50 , p<.001 , *r* = .81) condition. The proportion of correct responses per participant is shown in Figure 3. There was evidence against a main effect of condition ( ${\mathrm{BF}}_{10}=0.13$ [0.03, 0.46], β=0.02 , *SE* = 0.10, 95% CI = [–0.17, 0.21]) and evidence against to no evidence for a main effect of association (depending on the choice of prior, ${\mathrm{BF}}_{10}$ = 0.31 [0.07, 1.14], β=−0.15 , *SE* = 0.05, 95% CI = [–0.24, –0.06]). There was, depending on the choice of the prior, weak evidence against to weak evidence for an interaction of condition and association ( ${\mathrm{BF}}_{10}=1.16$ [0.29, 3.35], β=0.15 , *SE* = 0.06, 95% CI = [0.03, 0.28]). Post-hoc pairwise comparisons revealed that, in the agency condition, the association agent–non-agent was retrieved more likely than the association non-agent–non-agent (log odds ratio [log OR] = 0.15, 95% CI = [0.06, 0.24]), whereas memory performance for the two types of associations did not differ in the non-agency condition (log OR = 0.00, 95% CI = [–0.09, 0.08]).

#### Dependency

The dependency of the retrieval of event elements is shown in Figure 4. There was a significant positive dependency in both the agency condition (*D* = 0.07, *SE* = 0.01, *p* < .001) and the non-agency condition (*D* = 0.10, *SE* = 0.01, *p* < .001). Testing for a difference in dependency between conditions, the dependency in the agency condition was not significantly larger than the one in the non-agency condition ( $D_{\mathrm{diff}}=-0.03$ , *SE* = 0.02, *p* = .95).^17^

### Discussion

In Experiment 6, we aimed at increasing memory performance to be able to reliably detect differences in dependency between the experimental conditions and adjusted the agency manipulation to account for a potential confound (active vs passive sentence structure) in previous experiments. We again found a significant positive dependency in both conditions. However, the dependency in the agency condition was not significantly larger than the one in the non-agency condition. Descriptively, the results even pointed in the opposite direction. Thus, Hypothesis 1 was again not supported. This is despite us succeeding in increasing memory performance compared with Experiments 4 and 5. Although performance was still lower than in previous studies (Horner & Burgess, 2013; Schreiner et al., 2023), the given level of performance should have enabled us to detect a difference in dependency between conditions given our sample size. Thus, although we cannot completely rule out that differences in dependency may have been concealed due to low memory performance (cf. Schreiner & Meiser, 2023), Experiment 6 provides stronger evidence against the supposition that the presence of an agentic element in an event facilitates the binding of event elements.

## General discussion

In six experiments, we investigated whether the binding of event elements in episodic memory is influenced by agency, using a linguistic agency manipulation and the stochastic dependency of the retrieval of event elements as an indicator of binding effects. The results of this research yielded no evidence for a facilitating effect of agency on the binding of event elements. In addition, results strongly diverged between experiments in which event elements were presented sequentially pairwise or simultaneously, suggesting an effect of the experimental paradigm. An overview of the experimental setups and results is given in Table 1.

**Table 1.** Overview of methods aspects and results for all experiments.

	Experiment					
	1	2	3	4	5	6^a^
Encoding	Separated	Separated	Separated	Simultaneous	Simultaneous	Simultaneous
Design	Within	Within	Between	Between	Between	Between
Material	Objects	Objects	Animals	Animals	Animals	Animals
Event repetition	No	No	No	No	No	Yes
Memory test	Implicit	Implicit	Implicit	Implicit	Implicit	Intentional
Conditions	2	8	2	2	2	2
Events (total)^b^	48	48	24	24	24	24
Events (per condition)^b^	24	6	24	24	24	24
Learning trial duration	8 s	8 s	10 s	10 s	12.5 s	12.5 s
Data collection	Web	Web	Prolific	Prolific	Prolific	Prolific
*N*	39	242	59/58	122/117	180/171	161/164
*D* (*SE*) in agency condition	0.04 (0.02)	0.01 (0.02)	0.00 (0.02)	**0.04 (0.01)**	**0.07 (0.01)**	**0.07 (0.01)**
*D* (*SE*) in non-agency condition	0.01 (0.02)	0.02 (0.02)	0.03 (0.02)	0.02 (0.01)	**0.04 (0.01)**	**0.10 (0.01)**
*D*diff (*SE*)	0.03 (0.03)	–0.01 (0.02)	–0.03 (0.03)	0.02 (0.03)	0.03 (0.02)	–0.03 (0.02)

*D*: dependency of the retrieval of event elements; *D*diff: difference in dependency of the retrieval of event elements between conditions.

Significant results are set in boldface. Learning trial duration encompasses the fixation cross, the sentence presentation, and the blank screen. Sample sizes divided by a slash (/) refer to the sample size in the agency condition and non-agency condition, respectively (for between-subjects designs). For Experiment 2, result information refers to the closed-loop conditions.

a In Experiment 6, we adapted the agency manipulation and it thus slightly differed from the one in Experiments 1–5.

b Not including primacy buffers.

### Free association may facilitate binding across temporarily divided encoding episodes

We only found significant dependencies of the retrieval of event elements when using the simultaneous encoding paradigm (Horner & Burgess, 2013), in which event elements are presented simultaneously (Experiments 4–6), but not when using the separated encoding paradigm (Horner & Burgess, 2014; Horner et al., 2015), in which event elements are presented sequentially pairwise (Experiments 1–3, see Supplementary Appendix). This differs from previous findings, which showed significant dependencies of the retrieval of event elements also in the separated encoding paradigm, at least for coherent encoding episodes in which all possible pairwise associations are presented (Bisby et al., 2018; Horner & Burgess, 2014; Horner et al., 2015; Joensen et al., 2020; Schreiner et al., 2023). Some studies even found comparable dependencies between separated and simultaneous encoding conditions (Bisby et al., 2018; Horner & Burgess, 2014). However, in all of these studies event elements were presented as individual words or images, whereas we presented event elements embedded in sentences. In addition, our event elements were more semantically related, all of them being either objects or animals, than in the other studies in which event elements belonged to more distinct categories (e.g., animals, objects, and locations). In all of the studies, participants were required to imagine the event elements as part of a scene and imagine them interacting in a meaningful manner. This may be easier if event elements are presented as individual words or images and are more semantically distinct because this allows participants to more freely associate them than if they are presented in a more guided manner and are more semantically similar. When freely associating event elements, participants may also try to construct scenes that make sense to them, whereas the sentences presented in our experiments may have made less sense to the participants. Thus, the free association of event elements may facilitate the formation of coherent memory representations, whereas a more prestructured presentation of events may have caused participants to rely on independent pairwise representations. This adds to previous research suggesting that additional processes are required when binding event elements across temporarily divided encoding episodes compared with binding them within a single encoding episode (James et al., 2020). Besides written (rather than pictorial) and unidimensional (rather than multidimensional) presentation of event elements (James et al., 2020), the opportunity to freely associate them may facilitate binding. Thus, the agency manipulation we employed, in which event elements are embedded in sentences, may not work well in combination with the separated encoding paradigm used in Experiments 1–3.

### No evidence for facilitating effects of agency on the binding of event elements

When using the simultaneous encoding paradigm, we found significant dependencies in event element retrieval. In Experiment 4, this was only the case in the agency condition. In Experiments 5 and 6, we found significant dependencies in both the agency and non-agency condition. However, the difference in dependency between conditions was not significant, yielding no evidence for a facilitating effect of agency on the binding of event elements. Memory performance in Experiments 4 and 5 was rather poor, which may have concealed a potential effect of agency, since it is harder to find differences in dependency between conditions at lower levels of memory performance (cf. Schreiner & Meiser, 2023). However, in Experiment 6, we managed to increase performance, while controlling for a potential confound in Experiments 4 and 5 (the use of active vs passive sentence structures in the agency and non-agency condition, respectively) and the results still suggested no facilitating effect of agency. Thus, the results of this research suggest that the presence of an agentic element in an event does not facilitate the binding of event elements in episodic memory. Even if one assumes that the presence of an agentic element does actually facilitate the binding of event elements, the current findings may indicate that such an effect is probably rather weak and can likely not fully account for previously found facilitating effects of animacy on the binding of event elements (Schreiner et al., 2023).

This research extends previous findings on effects of agency in relation to memory and cognition. Although agency has been found to influence visual attention (Huffman & Brockmole, 2020; Wen & Haggard, 2018), object vision (Haxby et al., 2020; Thorat et al., 2019), face perception (Walker & Keller, 2019), autobiographical memory (Woike et al., 1999; Woike & Polo, 2001), hippocampal responses to expectation violations in episodic memory (Jainta et al., 2022), and memory performance (Hon & Yeo, 2021; Hornstein & Mulligan, 2001), this research suggests that effects of agency, at least as part of the stimulus, on cognition do not or only weakly extend to the formation of memory representations in episodic memory by influencing binding processes. Importantly, we focused on agency as part of an (external) stimulus, whereas the majority of previous research has focused on the agency of the participant (e.g., see the enactment effect, Engelkamp, 1986; Hornstein & Mulligan, 2001; Roberts et al., 2022). This research did not involve enactment, and participants may be considered observers of the to-be-imagined events. Therefore, this research does not preclude that agency on the participants’ side may facilitate the binding of event elements in episodic memory. Indeed, there is preliminary evidence for such an effect (Ruiz et al., 2023). This may suggest that some degree of self-reference (cf. Sui & Humphreys, 2015; Symons & Johnson, 1997) or personal relevance (cf. Nairne et al., 2017) may be necessary for effects of agency to emerge. In this research, participants likely took the role of a passive observer of the described events. Effects may possibly emerge if participants are more involved in an event, such that the action of the agent has some bearing on themselves.

### Limitations

There are at least four limitations concerning this research. First, all event elements in our experiments were either objects or animals, belonging to different subcategories of these classes (e.g., tools or mammals). We did this to control for potential confounding effects of animacy (cf. Schreiner et al., 2023). However, a drawback of using stimuli from the same superordinate category is that they are more semantically similar than when using stimuli from different categories (e.g., animals and locations). Thus, the stimuli in our experiments may have been harder to discriminate than stimuli used in previous studies (Bisby et al., 2018; Horner & Burgess, 2013, 2014; Horner et al., 2015; James et al., 2020; Joensen et al., 2020; Schreiner et al., 2023), which may have affected the results. In addition, not all the animal subcategories we used may be perceived as equal regarding animacy. Insects may be associated with lower animacy than birds or mammals (Connolly et al., 2012; Sha et al., 2015; Thorat et al., 2019). Thus, in Experiments 3–6, there may still have been some confounding with animacy, although it was certainly lower than if we had used more discrete categories such as animals, objects, and locations. Using animals from the same subcategory, however, would have further increased the semantic similarity of the stimuli and further decreased their discriminability. In addition, the results of the memory performance analyses indicated no difference in memory performance for the different subcategories, which makes effects of the study material seem unlikely, at least regarding memory performance.

Second, memory performance in the experiments was rather low, to which a reduced stimulus discriminability may have contributed due to using stimuli from the same superordinate category. Lower memory performance is associated with lower power for detecting dependencies and differences in dependency between conditions (Schreiner & Meiser, 2023). However, in Experiment 6, our measures for increasing memory performance proved successful, although performance was still lower than in previous studies (Horner & Burgess, 2013; Schreiner et al., 2023). Thus, although the results of Experiment 6 reduce this probability, we can not completely rule out that effects may have been masked due to low memory performance in our experiments.

Third, although there was no explicit agent in the non-agency condition of Experiments 1–5 (i.e., the agent was unknown), one may argue that participants may have imagined an agent and thus that there may have been an implicit agentic element in the non-agency condition. While this is a possibility, it seems inconsistent with the descriptive result patterns of Experiments 4 and 5 and the results of the aggregate dependency analysis, since, given a facilitating effect of agency on the binding of event elements, this should have boosted dependency in the non-agency condition. Rather, this line of reasoning suggests that effects of agency may actually be underestimated in these experiments, if participants indeed imagined additional agentic elements in the non-agency condition. However, the imagined agent may serve as an additional element, potentially increasing the amount of information in the non-agency compared with the agency condition. This increased amount of information may make binding in the non-agency condition more challenging. In Experiment 6, however, this is not an issue, because there was an explicit “agentic” element in the non-agency condition, which performed a passive activity next to the non-agentic elements. Although in this experiment the dependency in the non-agency condition was descriptively higher than in the agency condition, which may be considered as support for the claim that an imagined agent increases information load, this difference was non-significant.

Finally, all experiments were conducted online, with convenience Web samples for Experiments 1 and 2 and crowdsourced samples using Prolific for Experiments 3–6. Although web-based studies do not allow for the same degree of experimental control as do studies in the laboratory, several studies yielded comparable data quality for web- and lab-based studies (Armitage & Eerola, 2020; Bartneck et al., 2015; Dandurand et al., 2008; de Leeuw & Motz, 2016; Hilbig, 2016) and participants’ attention does not necessarily decrease during web-based studies (Clifford & Jerit, 2014; Hauser & Schwarz, 2016). We also employed rigourous data quality checks in our experiments. In addition, replicable effects for research on the binding of event elements have been found in web-based studies before (James et al., 2020; Schreiner et al., 2023). Thus, it is unlikely that the web-based setting of our experiments invalidates our results. Conducting the experiments online also allowed us to gather more diverse samples than in typical psychological lab-based studies (see also M. H. Birnbaum & Reips, 2005; Mason & Suri, 2012).

### Directions for future research

Because we cannot completely rule out that agency effects in this research may have been concealed due to low memory performance in the experiments, the question whether agency facilitates the binding of event elements in episodic memory requires further investigation. To this end, future research should try to replicate our findings using different agency manipulations than the linguistic manipulation used in this research. A different agency manipulation may also work in combination with the separated encoding paradigm (Horner & Burgess, 2014; Horner et al., 2015), allowing to investigate the structure in which event elements are bound together (see Experiment 2 in the Supplementary Appendix and Schreiner et al., 2023). Future research should aim at further boosting memory performance, since higher memory performance compared with this research would make it easier to detect effects (cf. Schreiner & Meiser, 2023). The possibility that free association facilitates the binding of event elements across temporarily divided encoding episodes should be directly tested in future research. Finally, as this research does not preclude effects of the agency of the participant, investigating such effects may be an interesting prospect for future research.

## Conclusion

In six experiments, we tested whether the presence of an agentic element in an event facilitates the binding of event elements in episodic memory. Our results do not suggest that the presence of an agentic element in an event facilitates binding. Such agency effects are thus likely not able to account for previously found effects of animacy on the binding of event elements (Schreiner et al., 2023). In addition, contrary to previous findings (Bisby et al., 2018; Horner & Burgess, 2014; Horner et al., 2015; Joensen et al., 2020; Schreiner et al., 2023), we only found binding effects when presenting event elements simultaneously, but not when presenting them sequentially pairwise. Given the different presentation format of events in this research compared with previous studies, this finding suggests that the opportunity to freely associate event elements may facilitate binding across temporarily divided encoding episodes.

## Supplemental Material

sj-pdf-1-qjp-10.1177_17470218231203951 – Supplemental material for Agency effects on the binding of event elements in episodic memory Supplemental material, sj-pdf-1-qjp-10.1177_17470218231203951 for Agency effects on the binding of event elements in episodic memory by Marcel R Schreiner, Arndt Bröder and Thorsten Meiser in Quarterly Journal of Experimental Psychology

## Notes

1. We increased the assumed baseline event-specific trait variance from 1 in Experiments 1–3 to 2 in Experiment 4 because of the change to simultaneous event element presentation, for which higher dependencies may be expected. Previous empirical data based on the simultaneous encoding paradigm yielded higher event-specific trait variances (James et al., 2020; Ngo et al., 2019; see also Schreiner et al., 2023). A higher baseline event-specific trait variance is associated with reduced power for detecting differences between conditions (see Schreiner & Meiser, 2023) and thus increases the demand in sample size.

2. Due to a participant error on Prolific the data of one additional participant were collected.

3. We did not exclude participants who gave invalid exclusion reasons such as subjective bad memory performance.

4. The sample was not age-limited. Although binding effects may decrease in old age (Naveh-Benjamin, 2000; Ngo & Newcombe, 2021; Old & Naveh-Benjamin, 2008), effects still emerge in healthy older adults. In addition, the number of older participants was very low in the experiments (e.g., only up to five participants were older than 60 years in the different experiments). Therefore, it is unlikely that the age of the participants substantially affected the results.

5. The insects stimuli contained some animals that are not actually insects but are commonly perceived as such (e.g., spider). They belong to the more general category of arthropods.

6. Note that, for assessing dependencies of the retrieval of event elements, events with at least three elements need to be tested (see Horner & Burgess, 2013).

7. This substituted the attention checks used in Experiments 1 and 2. The number of participants taking more than 10 s to click on any of the continue buttons was quite low (3 in Experiment 4, and 12 in Experiment 5 [one of which was excluded due to a very long response time]). In addition, we checked for conspicuous response patterns during the test phase.

8. This initial presentation of the cue was intended to improve data quality by preventing participants from clicking through the test phase and make them engage in active retrieval attempts.

9. We used distractors from other events to ensure that all possible response options were equally familiar. Using distractors that were never presented during the experiment would confound memory for individual event elements and memory for tested associations.

10. Association refers to the element pair being tested. There were three associations tested in each condition (i.e., *agent–non-agent_1_, agent–non-agent_2_, and non-agent_1_–non-agent_2_* in the agency condition, and mammal–bird, mammal–insect, and bird–insect in the non-agency condition). Associations do not distinguish between the direction of testing (e.g., the cue–target pairs bird–insect and insect–bird in the non-agency condition both test the association bird–insect).

11. Although the item content could vary across participants, as events were randomly generated for each participant, items were ordered by event number and cue type for the analysis. Thus, for example, for participants in the agency condition, the first item reflects the outcome of the test trial in which the agentic element served as the cue regarding the first presented event. Due to the nature of the items, person and item intercepts were entered as crossed random effects into the models.

12. Item difficulty was estimated for each item.

13. Although we implemented an incidental memory test in the previous experiments, we switched to an intentional memory test in Experiment 6 because knowledge about a later memory test may cause participants to exert more effort and pay more attention during the learning phase, thereby increasing memory performance.

14. We switched some verbs used as primacy buffers and some verbs used as main material.

15. For emotionality, we recoded the values to reflect the absolute distance from the neutral point of the scale (50).

16. As can be expected from previous studies (Halicki et al., 2021; Henley et al., 1995; Lowder & Gordon, 2015), mean ratings of agency and activity were strongly correlated, *r* = .92, *SE* = 0.06, *t*(25) = 16.52, *p* < .001.

17. Testing in the opposite direction also yielded a non-significant result (*p* = .05).
