# Supplementary material for: Agency effects on the binding of event elements in episodic memory
Source: Q J Exp Psychol (Hove). 2023 Oct 31;77(6):1201–20. doi: 10.1177/17470218231203951 (PMC11134989; doi:10.1177/17470218231203951)
Supplement: sj-pdf-1-qjp-10.1177_17470218231203951 – Supplemental material for Agency effects on the binding of event elements in episodic memory [file sj-pdf-1-qjp-10.1177_17470218231203951.pdf]

Supplementary Appendix

Agency Effects on the Binding of Event Elements in Episodic Memory

Marcel R. Schreiner, Arndt Bröder, and Thorsten Meiser
Department of Psychology
School of Social Sciences
University of Mannheim

Supplementary Appendix

Agency Effects on the Binding of Event Elements in Episodic Memory

Experiment 1

In Experiment 1 we investigated whether the presence of an agentic element in an event facilitates binding. We expected to find a stronger stochastic dependency of the retrieval of event elements for events with an agentic element than for events without an agentic element (Hypothesis 1). The experiment's design, hypothesis, and analysis plan were preregistered at https://osf.io/kts8p.

Methods

Participants

Participants were recruited from the Web (using various channels such as Social Media, mailing lists, blogs, and the online research platform SurveyCircle, 2021). They could join a lottery for winning vouchers of a total value of 80€ or receive course credit. An a priori power analysis with simulated data based on data by Schreiner et al. (2023) for detecting a small to medium difference between conditions (difference in event-specific trait variances of 0.75 according to the statistical procedure [see the main article], cf. Glas et al., 2000; Wang et al., 2002, assumed baseline event-specific trait variance of 1) with 80% power using one-tailed testing yielded a desired sample size of 40 participants. Due to the potential necessity of some data exclusion we increased the desired sample size by 20%, thus planning for a sample size of 48 participants. Because we needed to exclude more participants than anticipated we collected data from an additional 15 participants. Thus, we collected data of 63 participants. All participants provided online informed consent for their participation and publication of their data. We excluded 20 participants from the analyses because they did not pass both attention checks. Another three participants were excluded because they conducted the study on a smartphone on which a correct display of the experiment content could not be guaranteed. An additional participant was excluded because their browsing behavior suggested they interrupted the experiment frequently and

for a longer duration. Thus, the final sample consisted of 39 participants (30 [77%] female, 32 [82%] students) with an average age of 29.6 years (*SD* = 9.8, range = 20-64). All participants indicated speaking German as their first language or fluently.

Design

The experiment employed a one-factorial (agency condition: agency vs. non-agency) within-subjects design. In the agency condition one event element served as the agent and was placed as the grammatical subject in active sentences. In the non-agency condition there was no agent and only passive sentences were used.

Material

Stimuli consisted of 144 German nouns representing three different stimulus types (common objects): 48 means of transportation (e.g., *bicycle*), 48 tools (e.g., *hammer*), and 48 foods (fruits and vegetables, e.g., *apple*). In addition, 48 verbs (e.g., *grab*) were used. An additional 24 nouns, 8 of each type, and 8 verbs were used as primacy buffers. Stimuli were partly taken from Schreiner et al. (2023). We used three types of objects to avoid confounding with animacy (cf. Schreiner et al., 2023). Using the stimuli, we randomly created 48 events for each participant, each consisting of a means of transportation, a tool, a food, and a verb. Events were randomly assigned to the 2 experimental conditions, resulting in 24 events per condition and 4 primacy buffer events per condition, which were presented first.

Procedure

The procedure was based on the separated encoding paradigm (Horner et al., 2015; Horner & Burgess, 2014). In the learning phase of the experiment, participants were presented a sentence containing two event elements and the verb associated with the event in each trial. There were three learning trials (i.e., sentences) for each event. Sentences referring to the same event were presented interleaved with sentences referring to other events. The presentation order was randomized with the constraint of a minimum of two other-event trials being presented between two same-event trials. The experimental

conditions were randomly distributed across learning trials. In the agency condition active sentences were used if the sentence contained the agent and passive sentences were used if it did not (e.g., *The bicycle grabs the hammer.*, *The bicycle grabs the apple.*, and *The hammer and the apple are being grabbed.*). Each stimulus type (i.e., means of transportation, tool, or food) served as the agent equally often across events. In the non-agency condition only passive sentences were used (e.g., *The bicycle and the hammer are being grabbed.*, *The bicycle and the apple are being grabbed.*, and *The hammer and the apple are being grabbed.*). In passive sentences it was randomized which of the event elements appeared in the first sentence position. Thus, all possible pairwise associations between event elements were shown across the three learning trials referring to the same event. Encoding episodes were thus coherent (using closed-loop structures, cf. Horner et al., 2015; Horner & Burgess, 2014). Event elements in the agency condition were defined in terms of whether they were the agent or one of the non-agents in an event, yielding the associations agent – non-agent$_1$, agent – non-agent$_2$, and non-agent$_1$ – non-agent$_2$. Event elements in the non-agency condition were defined in terms of their stimulus type, yielding the associations means of transportation – tool, means of transportation – food, and tool – food. Each trial consisted of a 0.5-s fixation cross, a 6-s sentence presentation, and a 1.5-s blank screen. The experiment included two attention checks. After 50% of learning trials (not counting primacy buffers) participants were asked to click on a continue button within 10 s and after 50% of test trials they were asked to select the top left response option. Other than that, the procedure was identical to the one of Experiment 4, described in the main article.

Data Analysis

Data analysis was identical to the one conducted in Experiment 4, described in the main article, with the following exceptions: For the exploratory analysis of memory performance, agency condition was used as a between- instead of a within-subjects factor in the Bayesian generalized linear mixed models. Consistent with our approach in Experiment 4 we coerced the associations agent – non-agent$_1$ and means of transportation

– tool, agent – non-agent$_2$ and means of transportation – food, and non-agent$_1$ – non-agent$_2$ and tool – food into a common factor level, respectively. For the dependency analysis, we fit a joint IRT model to the data of both agency conditions, since agency was manipulated within-subjects.

Results

Memory Performance

On average, the proportion of correct responses was $M = 0.30$ ($SD = 0.46$) in the agency condition and $M = 0.27$ ($SD = 0.44$) in the non-agency condition. Performance was significantly above chance in both the agency ($V = 666.00$, $p < .001$, $r = 0.76$) and non-agency ($V = 668.00$, $p < .001$, $r = 0.77$) condition. Figure S.1 shows a raincloud plot (Allen et al., 2021) of the proportion of correct responses per participant. There was, depending on the choice of prior, no evidence against to moderate evidence for a main effect of condition ($BF_{10} = 1.63$ [0.42, 5.26]), but the 95% credible interval did not include zero, suggesting that memory performance was lower in the non-agency condition than in the agency condition ($\beta = -0.23$, $SE = 0.11$, 95% CI = [-0.44, -0.01]). There was evidence against a main effect of association ($BF_{10} = 0.006$ [< 0.001, 0.08], agent–non-agent$_2$/transport–food: $\beta = -0.03$, $SE = 0.11$, 95% CI = [-0.24, 0.18], non-agent$_1$–non-agent$_2$/tool–food: $\beta = -0.02$, $SE = 0.11$, 95% CI = [-0.23, 0.19]) and against an interaction of condition and association ($BF_{10} = 0.03$ [0.002, 0.25], non-agency × agent–non-agent$_2$/transport–food: $\beta = 0.12$, $SE = 0.15$, 95% CI = [-0.18, 0.41], non-agency × non-agent$_1$–non-agent$_2$/tool–food $\beta = 0.07$, $SE = 0.15$, 95% CI = [-0.23, 0.37]).

Dependency

The dependency of the retrieval of event elements is shown in Figure S.2. The dependency in both the agency condition ($D = 0.04$, $SE = 0.02$, $p = .05$) and non-agency condition ($D = 0.01$, $SE = 0.02$, $p = .61$) was non-significant. The dependency in the agency condition was not significantly larger than the one in the non-agency condition

Figure S.1

Raincloud Plot Depicting the Proportion of Correct Responses per Participant by Agency Condition in Experiments 1-3

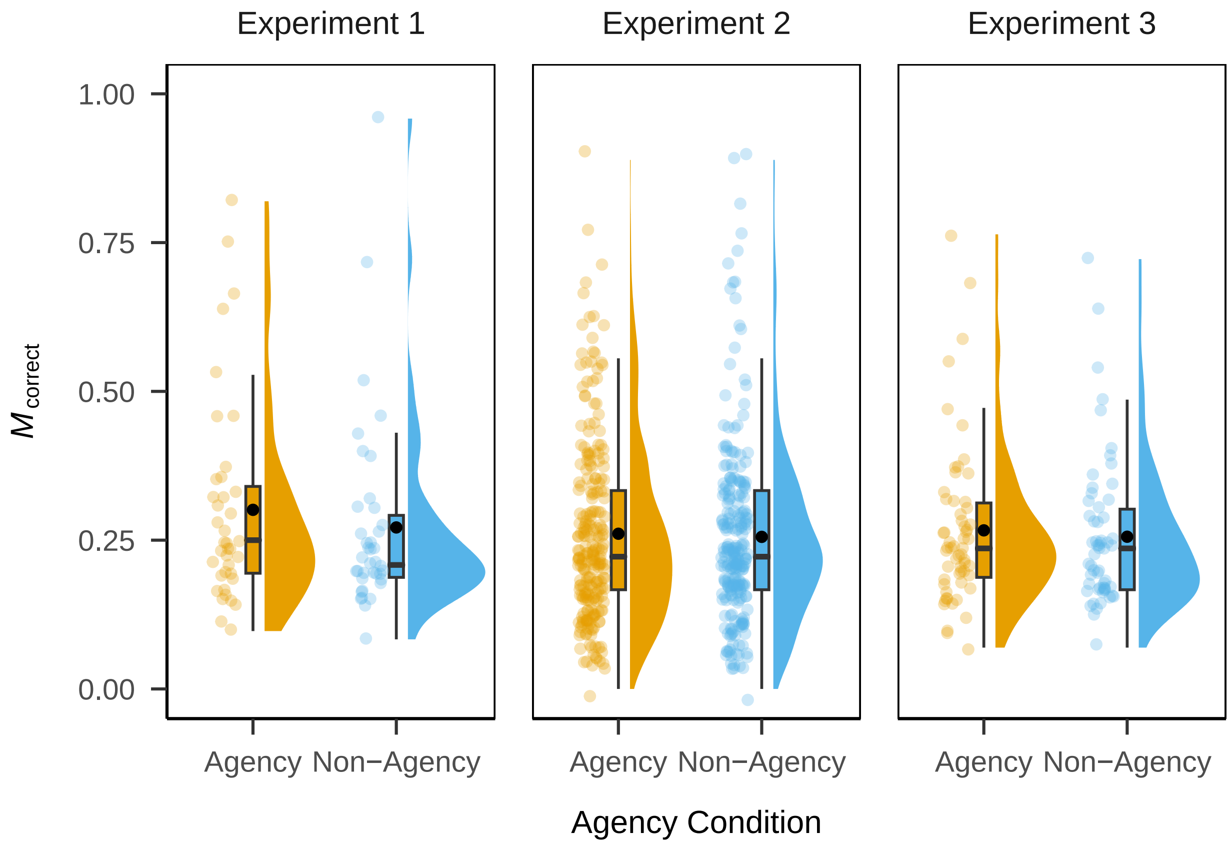

Notes. For Experiment 2 only data for the closed-loop conditions are shown. Black dots depict the mean across participants.

($D_{\text{diff}} = 0.03$, $SE = 0.03$, $p = .13$).

Discussion

In Experiment 1 we tested Hypothesis 1, which states that there is a stronger stochastic dependency of the retrieval of event elements for events with an agentic element than for events without an agentic element. There was no significant dependency in both the agency and non-agency condition and thus, the results are not informative regarding the hypothesis. However, descriptively, there was a positive dependency in the agency

Figure S.2

Dependency of the Retrieval of Event Elements by Agency Condition in Experiments 1-3

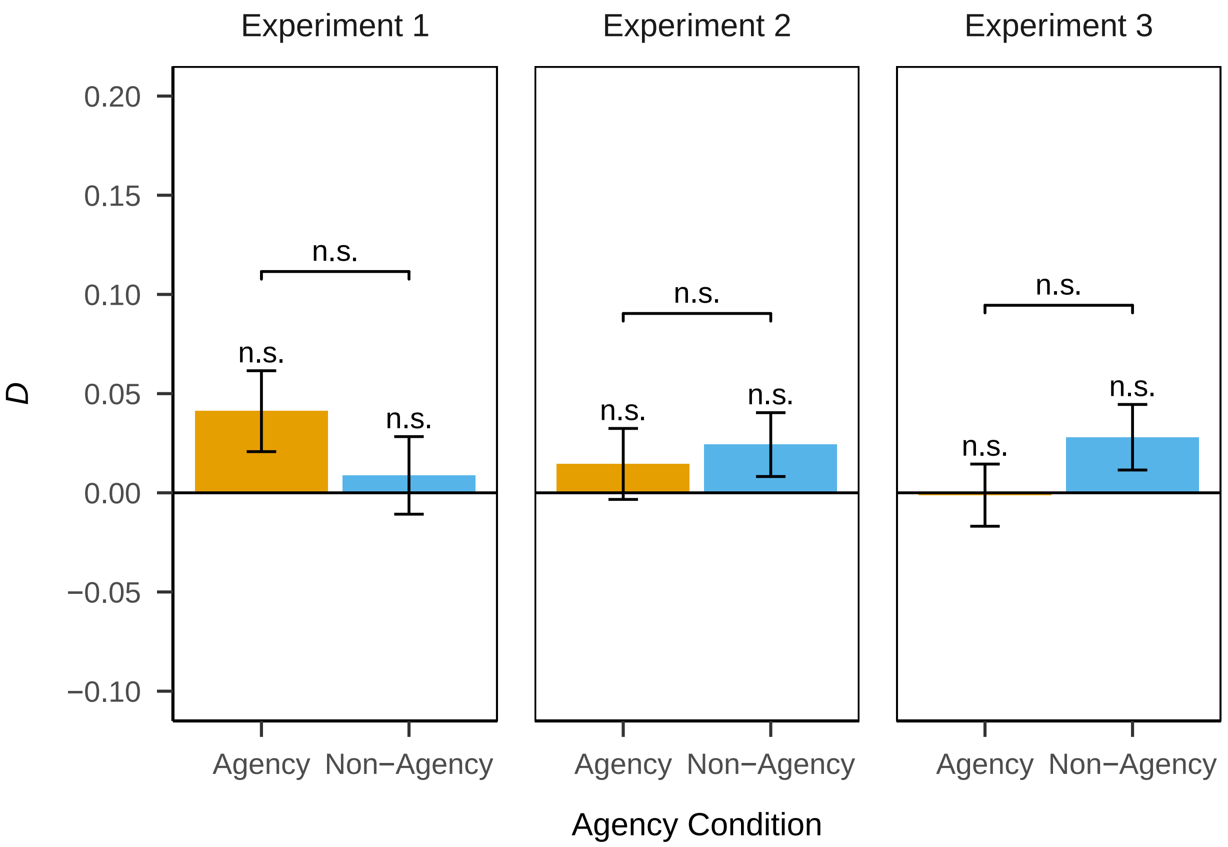

Notes. n.s = non-significant. Error bars represent ± *SE*. For Experiment 2 only data for the closed-loop conditions are shown.

condition that was larger than the dependency in the non-agency condition. Thus, the non-significant findings may be a Type II error or the effects may be rather small. In Experiment 2 we aimed for a larger sample size and thus for a higher power for detecting dependencies.

Experiment 2

In Experiment 2 we again investigated whether there is a stronger stochastic dependency of the retrieval of event elements for events with an agentic than for events without an agentic element (Hypothesis 1). In addition, we investigated the binding

structure of event elements by testing an integrated against a hierachical binding structure. To this end, we extended the experimental design to include non-coherent encoding episodes (open-loop structures, see Horner et al., 2015; Horner & Burgess, 2014) in addition to the coherent encoding episodes (closed-loop structures) that were used in Experiment 1. While in closed-loop structures all possible pairwise associations between event elements are shown, we consistently excluded specific associations from presentation in the open-loop structures (cf. Schreiner et al., 2023). Integrated and hierarchical binding structures make different predictions regarding the pattern of dependency across open-loop structures in which different associations are excluded. An integrated binding structure suggests that dependency does not vary as a function of the excluded association, because event elements are stored in a unitary representation that is accessible in an all-or-none manner (e.g., Damasio, 1989; Marr, 1971; Tulving, 1983). Thus, the association that is not presented should either be readily retrieved with the other associations (and thus dependency should be the same no matter which association is excluded) or the non-coherence of the encoding episodes may prevent integration and there should be no dependency in all open-loop conditions (and thus also no variation across open-loop conditions). A hierarchical binding structure suggests a system of pairwise bindings (see e.g., Cohen & Eichenbaum, 1993; Eichenbaum, 1999; Healy & Caudell, 2019) with asymmetrical binding strengths, in which event elements are preferentially bound to particular types of elements, such that some bindings are systematically prioritized over others (see Schreiner et al., 2023). Thus, excluding a more critical association should more strongly diminish dependency than excluding a less critical association, and consequently dependency should vary as a function of the excluded association. We expected that event elements are preferentially bound to the agent of the event, and thus expected to find a stronger stochastic dependency of the retrieval of event elements when excluding associations not involving an agentic element than when excluding associations involving an agentic element in non-coherent encoding episodes (Hypothesis 2). For events without

an agentic element we expected no differences in the stochastic dependency of the retrieval of event elements in non-coherent encoding episodes (Hypothesis 3), because in this case all associations should be equally important. The experiment's design, hypotheses, and analysis plan were preregistered at https://osf.io/kts8p.

Methods

Participants

Participants were again recruited from the Web, using the same channels as in Experiment 1. They could join a lottery for winning vouchers of a total value of 470€ or receive course credit. An a priori power analysis with simulated data for detecting the predicted results pattern with small to medium differences between conditions (difference in event-specific trait variances of 0.75 according to the statistical procedure, cf. Glas et al., 2000; Wang et al., 2002, assumed baseline event-specific trait variance of 1) with 80% power using one-tailed testing yielded a desired sample size of 240 participants. Due to the potential necessity of some data exclusion we increased the desired sample size by 20%, thus planning for a sample size of 288 participants. Because we needed to exclude more participants than anticipated we collected data from an additional 90 participants. Thus, we collected data of 378 participants. All participants provided online informed consent for their participation and publication of their data. We excluded three participants from the analyses because they indicated not speaking German fluently. Another 89 participants were excluded because they did not pass both attention checks. Another seven participants were excluded because they suggested their data should not be used for the study (e.g., due to distractions)[1]. Another 18 participants were excluded because they conducted the study on a smartphone on which a correct display of the experiment content could not be guaranteed. Another six participants were excluded because they indicated having recently participated in a similar study (i.e., Experiment 1). An additional 13 participants were

[1] The exclusion reason of one of the participants suggested that the participant may not want their data to be used. We excluded the data of this participant from the data we made publicly available.

excluded because their browsing behavior suggested they interrupted the experiment frequently and for a longer duration. Thus, the final sample consisted of 242 participants (181 [75%] female, 1 [0.4%] non-binary, 198 [82%] students) with an average age of 27.1 years (SD = 9.6, range = 18-68).

Design

The experiment employed a 2 (agency condition: agency vs. non-agency) × 4 (loop condition: closed-loop and three open-loops) within-subjects design. The closed-loop conditions (CL) were identical to the conditions in Experiment 1. In each of the three open-loop conditions we consistently excluded one pairwise association from presentation. Thus, in the agency condition there was one condition in which the association agent – non-agent$_1$ was excluded (OL-AgNAg$_1$), one condition in which the association agent – non-agent$_2$ was excluded (OL-AgNAg$_2$), and one condition in which the association non-agent$_1$ – non-agent$_2$ was excluded (OL-NAg$_1$NAg$_2$). In the non-agency condition there was one condition in which the association means of transportation – tool was excluded (OL-TrTo), one condition in which the association means of transportation – food was excluded (OL-TrFo), and one condition in which the association tool – food was excluded (OL-ToFo). The open-loop conditions were equated to the closed-loop conditions regarding the number of event elements rather than the number of presented associations. Previous studies yielded similar results irrespective of whether conditions were equated regarding the number of event elements or the number of presented associations (Horner & Burgess, 2014; Joensen et al., 2020).

Material and Procedure

Stimuli were identical to the ones of Experiment 1. Events were randomly assigned to the eight experimental conditions, resulting in six events per condition and one primacy buffer event per condition. The experimental procedure was identical to the one of Experiment 1, except that open-loop conditions were included in addition to closed-loop conditions. While event presentation in the closed-loop conditions consisted of three

learning trials, event presentation in the open-loop conditions consisted of two learning trials. For the open-loop conditions, test trials included one trial per event with a cue-target pair that was not presented jointly in the learning phase (inference trials). However, the respective cue and target overlapped with a common event element and could thus be flexibly related to enable reconstruction of the association that was excluded from presentation in the learning phase.

Data Analysis

Data analysis was identical to the one of Experiment 1 with the following exceptions: For the exploratory analysis of memory performance, loop condition was included as an additional predictor. Thus, there were three possible two-way interactions and one possible three-way interaction. To investigate the three-way interaction we compared the full model including all main effects and interactions with a model containing all main effects and two-way interactions but no three-way interaction. Similar to the handling of the factor association we coerced loop conditions into common factor levels to jointly include them in the models for the analysis of memory performance. To further investigate interactions, we conducted post-hoc pairwise comparisons using the package *emmeans* (version 1.8.6, Lenth, 2022). We considered a difference to be substantial if the 95% credible interval (highest posterior density interval) does not include zero.

Results

Memory Performance

On average, the proportion of correct responses was $M = 0.24$ ($SD = 0.42$) in the agency condition and $M = 0.22$ ($SD = 0.42$) in the non-agency condition. Performance was significantly above chance in both the agency ($V = 22,562.00$, $p < .001$, $r = 0.65$) and non-agency ($V = 20,736.00$, $p < .001$, $r = 0.61$) condition. The proportion of correct responses by agency condition, loop condition, and association is shown in Figure S.3. For loop conditions CL, Figure S.1 additionally shows the proportion of correct responses per

Figure S.3

Proportion of Correct Responses by Agency Condition, Loop Condition, and Association in Experiment 2

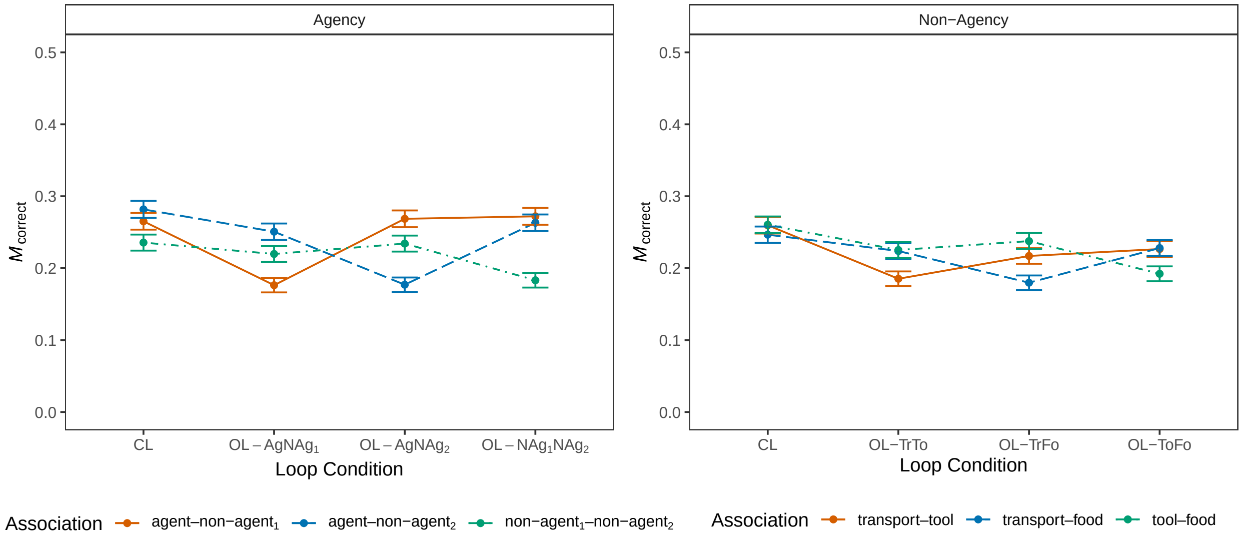

Notes. CL = closed-loop, OL-AgNAg$_1$ = open-loop with association agent – non-agent$_1$ excluded, OL-AgNAg$_2$ = open-loop with association agent – non-agent$_2$ excluded, OL-NAg$_1$NAg$_2$ = open-loop with association non-agent$_1$ – non-agent$_2$ excluded, OL-TrTo = open-loop with association means of transportation – tool excluded, OL-TrFo = open-loop with association means of transportation – food excluded, OL-ToFo = open-loop with association tool – food excluded, transport = means of transportation. Error bars represent ± *SEM*.

participant. There was, depending on the choice of prior, moderate evidence against to no evidence for a main effect of agency condition (BF_{10} = 0.47 [0.11, 1.83]), and the 95% credible interval included zero, suggesting that memory performance did not substantially differ between the agency and non-agency condition (β = -0.04, *SE* = 0.08, 95% CI = [-0.20, 0.13]). There was evidence for a main effect of loop condition ($BF_{10} > 1{,}000$ [> 1,000, > 1,000], OL-AgNAg$_1$/OL-TrTo: β = -0.54, *SE* = 0.09, 95% CI = [-0.72, -0.36], OL-AgNAg$_2$/OL-TrFo: β = 0.01, *SE* = 0.08, 95% CI = [-0.16, 0.18],

OL-NAg_1NAg_2/OL-ToFo:$\beta = 0.03$, $SE = 0.08$, 95% CI = [-0.14, 0.20]), but evidence against a main effect of association ($BF_{10} = 0.006$ [< 0.001, 0.10], agent–non-$agent_2$/transport–food: $\beta = 0.08$, $SE = 0.08$, 95% CI = [-0.08, 0.24], non-$agent_1$–non-$agent_2$/tool–food: $\beta = -0.17$, $SE = 0.08$, 95% CI = [-0.33, 0.00]). There was moderate evidence against to evidence for a two-way interaction of agency condition and association ($BF_{10} = 1.99$ [0.13, 16.82], non-agency × agent–non-$agent_2$/transport–food: $\beta = -0.14$, $SE = 0.12$, 95% CI = [-0.37, 0.09], non-agency × non-$agent_1$–non-$agent_2$/tool–food: $\beta = 0.17$, $SE = 0.12$, 95% CI = [-0.06, 0.40]). Post-hoc pairwise comparisons revealed that, in the agency-condition, memory performance was higher for associations involving the agent than for the association not involving the agent (log odds ratio [log OR] = 0.15, 95% CI = [0.06, 0.24] and log OR = 0.14, 95% CI = [0.05, 0.22]), whereas memory performance did not differ between the two associations involving the agent (log OR = 0.01, 95% CI = [-0.07, 0.10]). In the non-agency condition memory performance did not differ between associations. There was also evidence for a two-way interaction of loop condition and association ($BF_{10} > 1{,}000$ [> 1,000, > 1,000], OL-$AgNAg_1$/OL-TrTo × agent–non-$agent_2$/transport–food: $\beta = 0.38$, $SE = 0.12$, 95% CI = [0.14, 0.62], OL-$AgNAg_2$/OL-TrFo × agent–non-$agent_2$/transport–food: $\beta = -0.63$, $SE = 0.12$, 95% CI = [-0.87, -0.39], OL-NAg_1NAg_2/OL-ToFo × agent–non-$agent_2$/transport–food: $\beta = -0.12$, $SE = 0.12$, 95% CI = [-0.35, 0.11], OL-$AgNAg_1$/OL-TrTo × non-agent1–non-$agent_2$/tool–food: $\beta = 0.44$, $SE = 0.13$, 95% CI = [0.20, 0.69], OL-$AgNAg_2$/OL-TrFo × non-agent1–non-$agent_2$/tool–food: $\beta = -0.01$, $SE = 0.12$, 95% CI = [-0.25, 0.22], OL-NAg_1NAg_2/OL-ToFo × non-agent1–non-$agent_2$/tool–food: $\beta = -0.36$, $SE = 0.12$, 95% CI = [-0.60, -0.12]) that qualified the main effect of loop condition. Post-hoc pairwise comparisons revealed that memory performance was lower for associations that were not presented during the learning phase (i.e., to-be-inferred associations) than for associations that were presented during the learning phase (loop condition OL-$AgNAg_1$/OL-TrTo: log OR = -0.36, 95% CI = [-0.49, -0.23] and log OR =

-0.27, 95% CI = [-0.40, -0.13]; loop condition OL-AgNAg$_2$/OL-TrFo: log OR = 0.40, 95% CI = [0.27, 0.53] and log OR = -0.37, 95% CI = [-0.50, -0.24]; loop condition OL-NAg$_1$NAg$_2$/OL-ToFo: log OR = 0.38, 95% CI = [0.25, 0.51] and log OR = 0.36, 95% CI = [0.23, 0.49]). There was evidence against a two-way interaction of agency condition and loop condition (BF_{10} = 0.002 [< 0.001, 0.09], non-agency × OL-AgNAg$_1$/OL-TrTo: β = 0.09, *SE* = 0.13, 95% CI = [-0.16, 0.34], non-agency × OL-AgNAg$_2$/OL-TrFo: β = -0.25, *SE* = 0.12, 95% CI = [-0.49, -0.01], non-agency × OL-NAg$_1$NAg$_2$/OL-ToFo: β = -0.21, *SE* = 0.12, 95% CI = [-0.44, 0.03]) and against a three-way interaction (BF_{10} = 0.002 [< 0.001, 0.09], non-agency × OL-AgNAg$_1$/OL-TrTo × agent–non-agent$_2$/transport–food: β = -0.06, *SE* = 0.17, 95% CI = [-0.40, 0.27], non-agency × OL-AgNAg$_2$/OL-TrFo × agent–non-agent$_2$/transport–food: β = 0.44, *SE* = 0.17, 95% CI = [0.11, 0.78], non-agency × OL-NAg$_1$NAg$_2$/OL-ToFo × agent–non-agent$_2$/transport–food: β = 0.19, *SE* = 0.17, 95% CI = [-0.14, 0.52],non-agency × OL-AgNAg$_1$/OL-TrTo × non-agent1–non-agent$_2$/tool–food: β = -0.19, *SE* = 0.17, 95% CI = [-0.54, 0.14], non-agency × OL-AgNAg$_2$/OL-TrFo × non-agent1–non-agent$_2$/tool–food: β = 0.13, *SE* = 0.17, 95% CI = [-0.21, 0.46], non-agency × OL-NAg$_1$NAg$_2$/OL-ToFo × non-agent1–non-agent$_2$/tool–food: β = 0.12, *SE* = 0.17, 95% CI = [-0.22, 0.47]).

Dependency

The dependency of the retrieval of event elements in loop conditions CL is shown in Figure S.2. There was no significant dependency of the retrieval of event elements in any of the conditions. In loop condition CL, dependencies were D = 0.01 (*SE* = 0.02, p = .40) in the agency condition and D = 0.02 (*SE* = 0.02, p = .13) in the non-agency condition. The dependency in loop condition CL in the agency condition was not significantly larger than the one in the non-agency condition (D_{diff} = -0.01, *SE* = 0.02, p = .70). There was also no significant dependency in any of the open-loop conditions. In the agency condition, dependencies were D = 0.01 (*SE* = 0.02, p = .45) in loop condition OL-AgNAg$_1$, D = -0.02 (*SE* = 0.02, p = .13) in loop condition OL-AgNAg$_2$, and D = -0.01 (*SE* = 0.02, p =

.49) in loop condition OL-NAg_1NAg_2. In the non-agency condition, dependencies were $D =$ 0.02 (SE = 0.02, p = .29) in loop condition OL-TrTo, D = 0.02 (SE = 0.02, p = .20) in loop condition OL-TrFo, and D = 0.00 (SE = 0.02, p = .84) in loop condition OL-ToFo. The only significant dependency differences were a smaller dependency in loop condition OL-$AgNAg_2$ in the agency condition than in loop conditions CL (D_{diff} = -0.05, SE = 0.02, p = .02), OL-TrTo (D_{diff} = -0.04, SE = 0.02, p = .04), and OL-TrFo (D_{diff} = -0.05, SE = 0.02, p = .03) in the non-agency condition. All other dependency differences were non-significant ($p \geq .06$).

Discussion

In Experiment 2 we again tested Hypothesis 1. In addition, we tested an integrated against a hierarchical binding structure. Since we did not find a significant dependency in any of the experimental conditions, the results are uninformative regarding the hypotheses and cannot properly distinguish between an integrated and a hierarchical binding structure. It is noteworthy that memory performance was even lower than in Experiment 1 and memory performance in both Experiment 1 and 2 was lower than, for example, memory performance in the experiments by Schreiner et al. (2023). Since low memory performance is associated with lower power for detecting dependencies and differences in dependencies (Schreiner & Meiser, 2023), in Experiment 3 we made some changes to the experimental design and procedure intended to improve memory performance.

Experiment 3

In Experiment 3 we again investigated whether there is a stronger stochastic dependency of the retrieval of event elements for events with an agentic than for events without an agentic element (Hypothesis 1). We introduced a number of changes to the design and procedure of Experiment 1 (and the closed-loop conditions of Experiment 2) intended to improve memory performance to achieve higher power for detecting binding effects (cf. Schreiner & Meiser, 2023). We changed the experimental design from a within- to a between-subjects design while keeping the number of events per condition identical to

Experiment 1 (except for the primacy buffers). Thus, in total, participants saw only half the number of events as in Experiment 1 and consequently memory load was reduced. We further increased the presentation duration of the sentences. We also changed the stimuli from objects to animals, because using animate stimuli may lead to larger binding effects since they have an inherent agentic potential. This may also increase the plausibility of the described scenes and reduce processing costs due to semantic mismatches when combining the inherently non-agentic (or only weakly agentic) objects with an action verb (see Lowder & Gordon, 2015). The experiment's design, hypothesis, and analysis plan were preregistered at https://osf.io/vhmt4.

Methods

Participants

Participants were recruited via Prolific (https://www.prolific.co/) and received a compensation of £3.75 (£7.50/hour). They were prescreened to be native German speakers and to not conduct the study on a smartphone. An a priori power analysis with simulated data for detecting a medium difference between conditions[2] (difference in event-specific trait variances of 1 according to the statistical procedure, cf. Glas et al., 2000; Wang et al., 2002, assumed baseline event-specific trait variance of 1) with 80% power using one-tailed testing yielded a desired sample size of 100 participants (50 participants per between-subjects condition). Due to the potential necessity of some data exclusion we increased the desired sample size by 20% and collected data from 120 participants. All participants provided online informed consent for their participation and publication of their data. The data of one participant was not transmitted due to a technical error. Two participants were excluded because they suggested their data should

[2] We planned the sample size based on a larger effect in Experiment 3 than in the preceding experiments because we expected that the changes to the experimental design and procedure would lead to larger binding effects and we wanted to first evaluate the impact of the changes using a smaller sample so that we could later follow-up with a larger sample.

not be used for the study (e.g., due to tiredness). Thus, the final sample consisted of 59 participants in the agency condition (31 [53%] female, 2 [3%] non-binary, 21 [36%] students), with an average age of 34.9 years (SD = 13.2, range = 18-69), and 58 participants in the non-agency condition (25 [43%] female, 27 [47%] students), with an average age of 33.3 years (SD = 11.3, range = 19-62).

Design

The experiment employed a one-factorial (agency condition: agency vs. non-agency) between-subjects design. Participants were randomly assigned to the experimental conditions. Conditions were identical to the ones in Experiment 1 and to the closed-loop conditions in Experiment 2.

Material, Procedure, and Data Analysis

The material was identical to that of Experiment 4, described in the main article. The procedure was identical to the one of Experiment 1, except that we increased the duration of the sentence presentation during the learning phase to 8 s. Thus, a learning trial consisted of a 0.5-s fixation cross, an 8-s sentence presentation, and a 1.5-s blank screen (as was also the case in Experiment 4). In addition, we substituted the attention checks with a procedure intended to keep participants engaged during the learning phase — they were asked to click on a continue button after the primacy buffer trials and after 25%, 50%, and 75% of learning trials[3]. Data analysis was identical to the one conducted in Experiment 4, described in the main article. Fitting the bifactor IRT models again yielded some extreme estimates for item parameters that caused item responses in the simulated data used for the parametric bootstrap for testing differences between the experimental conditions to have no variance. As in Experiment 4, we substituted these parameters (two parameters [3%] in the model for the agency condition and seven parameters [10%] for the

[3] The number of participants taking more than 10 s to click on any of the continue buttons was quite low (this was only the case for 6 participants). In addition, we checked for conspicuous response patterns during the test phase.

model in the non-agency condition) with values randomly drawn from the empirical distribution of the remaining parameters.

Results

Memory Performance

On average, the proportion of correct responses was $M = 0.27$ ($SD = 0.44$) in the agency condition and $M = 0.26$ ($SD = 0.44$) in the non-agency condition. Performance was significantly above chance in both the agency ($V = 1,532.00$, $p < .001$, $r = 0.73$) and non-agency ($V = 1,140.00$, $p < .001$, $r = 0.68$) condition. The proportion of correct responses per participant is shown in Figure S.1. There was evidence against main effects of condition ($BF_{10} = 0.13$ [0.03, 0.48], β = -0.09, $SE = 0.14$, 95% CI = [-0.37, 0.19]) and association ($BF_{10} = 0.05$ [0.003, 0.66], agent–non-agent$_2$/mammal–insect: β = 0.02, $SE =$ 0.09, 95% CI = [-0.15, 0.19], non-agent$_1$–non-agent$_2$/bird–insect: β = -0.08, $SE = 0.09$, 95% CI = [-0.26, 0.09]) and against an interaction of condition and association ($BF_{10} =$ 0.02 [0.001, 0.22], non-agency $\times$ agent–non-agent$_2$/mammal–insect: β = 0.10, $SE = 0.12$, 95% CI = [-0.14, 0.35], non-agency $\times$ non-agent$_1$–non-agent$_2$/bird–insect: β = 0.01, $SE =$ 0.13, 95% CI = [-0.23, 0.26]).

Dependency

The dependency of the retrieval of event elements is shown in Figure S.2. The dependency in both the agency condition ($D = 0.00$, $SE = 0.02$, $p = .88$) and non-agency condition ($D = 0.03$, $SE = 0.02$, $p = .10$) was non-significant. The dependency in the agency condition was not significantly larger than the one in the non-agency condition ($D_{\text{diff}} = -0.03$, $SE = 0.03$, $p = .80$).

Discussion

In Experiment 3 we again tested Hypothesis 1. However, there was again no significant dependency in both the agency and non-agency condition and thus, the results are not informative regarding the hypothesis. In addition, the changes made to the

experimental procedure and design in Experiment 3 did not lead to an increase in memory performance compared to Experiments 1 and 2. It is possible that the more diverse sample counteracted effects of the changes of the experimental design and procedure. Compared to Experiments 1 and 2 the sample in Experiment 3 was older and comprised more males and fewer students.

References

Allen, M., Poggiali, D., Whitaker, K., Marshall, T. R., Langen, J. van, & Kievit, R. A. (2021). Raincloud plots: A multi-platform tool for robust data visualization. *Wellcome Open Research*, 4:63. https://doi.org/10.12688/wellcomeopenres.15191.2

Cohen, N. J., & Eichenbaum, H. (1993). *Memory, amnesia, and the hippocampal system.* MIT Press.

Damasio, A. R. (1989). The brain binds entities and events by multiregional activation from convergence zones. *Neural Computation*, *1*(1), 123–132. https://doi.org/10.1162/neco.1989.1.1.123

Eichenbaum, H. (1999). The hippocampus and mechanisms of declarative memory. *Behavioural Brain Research*, *103*(2), 123–133. https://doi.org/10.1016/S0166-4328(99)00044-3

Glas, C. A. W., Wainer, H., & Bradlow, E. T. (2000). MML and EAP estimation in testlet-based adaptive testing. In W. J. van der Linden & C. A. W. Glas (Eds.), *Computerized adaptive testing: Theory and practice* (pp. 271–287). Kluwer.

Healy, M. J., & Caudell, T. P. (2019). Episodic memory: A hierarchy of spatiotemporal concepts. *Neural Networks*, *120*, 40–57. https://doi.org/10.1016/j.neunet.2019.09.021

Horner, A. J., Bisby, J. A., Bush, D., Lin, W.-J., & Burgess, N. (2015). Evidence for holistic episodic recollection via hippocampal pattern completion. *Nature Communications*, *6*(1), 7462. https://doi.org/10.1038/ncomms8462

Horner, A. J., & Burgess, N. (2014). Pattern completion in multielement event engrams. *Current Biology*, *24*(9), 988–992. https://doi.org/10.1016/j.cub.2014.03.012

Joensen, B. H., Gaskell, M. G., & Horner, A. J. (2020). United we fall: All-or-none forgetting of complex episodic events. *Journal of Experimental Psychology: General*, *149*(2), 230–248. https://doi.org/10.1037/xge0000648

Lenth, R. (2022). *emmeans: Estimated marginal means, aka least-squares means. R package version 1.8.6.* https://cran.r-project.org/package=emmeans

Lowder, M. W., & Gordon, P. C. (2015). Natural forces as agents: Reconceptualizing the animate–inanimate distinction. *Cognition*, *136*, 85–90. https://doi.org/10.1016/j.cognition.2014.11.021

Marr, D. (1971). Simple memory: A theory for archicortex. *Philosophical Transactions of the Royal Society of London. B: Biological Sciences*, *262*(841), 23–81. https://doi.org/10.1098/rstb.1971.0078

Schreiner, M. R., & Meiser, T. (2023). Measuring binding effects in event-based episodic representations. *Behavior Research Methods*, *55*, 981–996. https://doi.org/10.3758/s13428-021-01769-1

Schreiner, M. R., Meiser, T., & Bröder, A. (2023). The binding structure of event elements in episodic memory and the role of animacy. *Quarterly Journal of Experimental Psychology*, *76*(4), 705–730. https://doi.org/10.1177/17470218221096148

SurveyCircle. (2021). *Research website SurveyCircle. Published 2016.* https://www.surveycircle.com.

Tulving, E. (1983). *Elements of episodic memory.* Oxford University Press.

Wang, X., Bradlow, E. T., & Wainer, H. (2002). A general Bayesian model for testlets: Theory and applications. *Applied Psychological Measurement*, *26*(1), 109–128. https://doi.org/10.1177/0146621602026001007
